# α,ω-Epoxide, Oxetane, and Dithiocarbonate Telechelic Copolyolefins: Access by Ring-Opening Metathesis/Cross-Metathesis Polymerization (ROMP/CM) of Cycloolefins in the Presence of Functional Symmetric Chain-Transfer Agents

**DOI:** 10.3390/polym10111241

**Published:** 2018-11-09

**Authors:** Elise Vanbiervliet, Stéphane Fouquay, Guillaume Michaud, Frédéric Simon, Jean-François Carpentier, Sophie M. Guillaume

**Affiliations:** 1 Univ Rennes, CNRS, ISCR (Institut des Sciences Chimiques de Rennes)—UMR 6226, F-35000 Rennes, France; elise.vanbiervliet@hotmail.fr; 2 BOSTIK S.A., 253, Avenue du Président Wilson, F-93211 La Plaine Saint-Denis, France; stephane.fouquay@bostik.com; 3 BOSTIK, ZAC du Bois de Plaisance, 101, Rue du Champ Cailloux, F-60280 Venette, France; Guillaume.MICHAUD@bostik.com (G.M.); frederic-n.simon@bostik.com (F.S.)

**Keywords:** chain-transfer agent, dithiocarbonate, epoxide, oxetane, functional polyolefin, ring-opening metathesis polymerization, telechelic polymer

## Abstract

Epoxide- and oxetane-α,ω-telechelic (co)polyolefins have been successfully synthesized by the tandem ring-opening metathesis polymerization (ROMP)/cross-metathesis (CM) of cyclic olefins using Grubbs’ second-generation catalyst (**G2**) in the presence of a bifunctional symmetric alkene epoxide- or oxetane-functionalized chain-transfer agent (CTA). From cyclooctene (COE), *trans*,*trans*,*cis*-1,5,9-cyclododecatriene (CDT), norbornene (NB), and methyl 5-norbornene-2-carboxylate (NB^COOMe^), with bis(oxiran-2-ylmethyl) maleate (CTA **1**), bis(oxetane-2-ylmethyl) maleate (CTA **2**), or bis(oxetane-2-ylmethyl) (*E*)-hex-3-enedioate (CTA **3**), well-defined α,ω-di(epoxide or oxetane) telechelic PCOEs, P(COE-*co*-NB or -NB^COOMe^)s, and P(NB-*co*-CDT)s were isolated under mild operating conditions (40 or 60 °C, 24 h). The oxetane CTA **3** and the epoxide CTA **1** were revealed to be significantly more efficient in the CM step than CTA **2**, which apparently inhibits the reaction. Quantitative dithiocarbonatation (CS_2_/LiBr, 40 °C, THF) of an α,ω-di(epoxide) telechelic P(NB-*co*-CDT) afforded a convenient approach to the analogous α,ω-bis(dithiocarbonate) telechelic P(NB-*co*-CDT). The nature of the end-capping function of the epoxide/oxetane/dithiocarbonate telechelic P(NB-*co*-CDT)s did not impact their thermal signature, as measured by DSC. These copolymers also displayed a low viscosity liquid-like behavior and a shear thinning rheological behavior.

## 1. Introduction

Telechelic polymers, i.e., polymers with functional end-groups, attract much interest due to their unique properties. They are commonly used as precursors to block copolymers, as cross-linking agents, or as intermediates in the formation of polymeric networks [1,2,3,4]. Polyolefins (POs) are the most commercially produced polymers, featuring great resistance to harsh chemical environments and broad-ranging mechanical and thermal characteristics, resulting in a wide variety of applications, such as in packaging, including food wrapping, electrical wires protection or piping systems, fabrics, automobiles, and also in the pharmaceutical and medical industries. Telechelic POs are valued synthetic targets with major uses that are derived from their adhesion, toughness, print/paintability, compatibility with diverse, more polar materials, and rheological properties. Efforts both in academia and in industry are, thus, currently dedicated to developing such polymer materials. However, their synthesis remains challenging. While post-polymerization chemical modification enables us to incorporate the desired functions at the termini of POs, metathesis polymerizations have been revealed to be a most useful and effective direct approach towards α,ω-functional POs [5,6,7,8,9,10,11,12,13].

As pioneered by Grubbs and Hillmyer, telechelic POs are nowadays most commonly and conveniently prepared by the simultaneous ring-opening methathesis polymerization (ROMP) and cross-metathesis (CM) polymerization of a cylic olefin using a metal transition alkylidene catalyst in the presence of a symmetric functional chain-transfer agent (CTA). Indeed, symmetric CTAs are more suitable for selectively preparing the corresponding α,ω-difunctional POs, as compared to the monofunctional asymmetric CTAs, which are well-known to give a statistical distribution of end-capped polymers from metathesis processes, including linear (isomerized) and/or cyclic nonfunctional, (isomerized) α-monofunctional, and/or α,ω-difunctional POs. A most efficient transition metal-alkylidene catalyst for this process is the Grubbs’ second generation ruthenium complex (**G2**). This catalyst remains highly reactive in the presence of large loadings of CTA, providing well-defined functional POs. Tandem ROMP/CM of various cyclic olefins have, thus, enabled the synthesis of a wide range of α,ω-difunctional POs featuring end-capping groups as diverse as acetoxy, carboxylate, halide, pseudo-halide, hydroxyl, and cross-linkable epoxide or methacrylate [14,15,16,17,18,19,20,21,22,23,24,25,26,27,28,29,30,31,32,33]. Recent advances in our group enlarged the family of telechelic (co)polyolefins ((co)POs) derived from methathesis routes to alkoxysilyl, azlactone, (thio)carbonate, and epoxide end-capped polymers [34,35,36,37,38,39,40,41,42]. We report herein on the synthesis and characterization of epoxide, oxetane, and dithiocarbonate α,ω-difunctional (co)POs through the ROMP/CM of some cyclic olefins, namely cyclooctene (COE), *trans*,*trans*,*cis*-1,5,9-cyclododecatriene (CDT), norbornene (NB), and methyl 5-norbornene-2-carboxylate (NB^COOMe^), using **G2** in the presence of the corresponding epoxide or oxetane functional symmetrical CTA. Prior investigations on the original oxetane end-capped poly(cyclooctene) (PCOE) are first presented, prior to focusing on alike copolymers.

## 2. Experimental Section

### 2.1. Materials

All catalytic metathesis experiments were performed under an inert atmosphere (argon) using standard Schlenk line and glove box techniques. Grubbs’ 2nd generation catalyst, ([(IMesH_2_)(Cy_3_P)RuCl_2_(=CHPh)], **G2**), Hoveyda–Grubbs’ 2nd generation catalyst ([(IMesH_2_)(Cy_3_P)RuCl_2_(=CHC_6_H_4_CMe_2_O)], **HG2**), (3-methyloxetane-3-yl)methanol, (3-methyloxetane-3-yl) ethanol, *Candida Antarctica* Lipase B (CALB), (*E*)-hex-3-enedioic acid, polyethyleneimine (Lupasol FG^®^, BASF, Levallois Perret, France), and all other reagents (Aldrich (Saint-Quentin Fallavier, France) or ACROS (Fontenay-sous-Bois, France), unless otherwise mentioned) were used as received. Cyclooctene (COE), *trans*,*trans*,*cis*-1,5,9-cyclododecatriene (CDT), and norbornene (NB) were purchased from Sigma-Aldrich or ACROS, and dried and distilled over CaH_2_ before use. CH_2_Cl_2_ (stabilized with amylene) was purified on an MBraun system over activated 3 Å molecular sieves. The bis(oxiran-2-ylmethyl) maleate CTA **1** was prepared according to the reported procedure upon epoxidation of diallyl maleate in the presence of metachloroperbenzoic acid (MCPBA) (CH_2_Cl_2_, reflux, 48 h; 48% yield) [39,43]. Diethyl fumarate (***i***) was prepared upon esterification of fumaric acid and ethanol in acidic conditions (reflux, 48 h) in the presence of APTS.

### 2.2. Instrumentation and Measurements

^1^H (500, 400 MHz) and ^13^C{^1^H} (125, 100 MHz) NMR spectra were recorded on Bruker Avance AM 500 and AM 400 spectrometers (Bruker, Billerica, MA, USA) at 23 °C in CDCl_3_ or DMSO-*d*_6_. Chemical shifts (δ) are reported in ppm and were referenced internally relative to tetramethylsilane (δ 0 ppm) using the residual ^1^H and ^13^C solvent resonances of the deuterated solvent.

Monomer conversions were determined from ^1^H NMR spectra of the crude polymer sample, from the integration (Int.) ratio Int._Polymer_/[Int._Polymer_ + Int._monomer_], using the methine hydrogens (–C*H*=C*H–*: δ 5.30, 5.66 (PCOE, COE); 5.44, 5.09 (PCDT, CDT); 5.31, 6.04 (PNB, NB); 5.36, 5.98 (PNB^COOMe^, NB^COOMe^).

**Figure 1:**
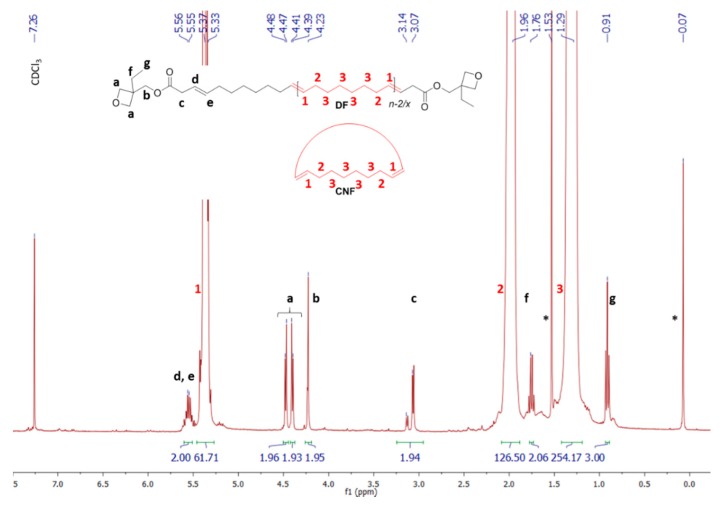
^1^H NMR spectrum (500 MHz, CDCl_3_, 25 °C) of an α,ω-dioxetane telechelic PCOE sample prepared by ring-opening metathesis polymerization (ROMP)/cross-metathesis (CM) of COE in the presence of **G2**/CTA **3** in CH_2_Cl_2_ (Table 1, entry 4) (*: residual solvents: δ (ppm) 1.59 H_2_O, 0.07 grease).

The molar mass values of PCOE samples were determined by a ^1^H NMR analysis in CDCl_3_ (*M*_n,NMR_) from the integral value ratio of the signals of end-groups’ hydrogens (typically δ ca. 3.07 ppm (H^c^)) to internal olefin hydrogens (δ ca. 5.35 (H^1^)) (Figure 1). For copolymers, the molar mass values were determined by a ^1^H NMR analysis according to: *M*_n,NMR_ = {*M*_NB_ × Int.(δH^6trans^ 2.79 + H^6cis^ 2.44)} + {*M*_CDT_ × Int.(δH^1,4^5.41)/3} + *M*_CTA**3**_, with the integral of the signal for the hydrogens adjacent to the internal C=C bond (δ 3.07 ppm H^c^) (Figure 3) arbitrarily set to the value of 2. The molar mass values of the NB/CDT copolymers were determined by a ^1^H NMR analysis in CDCl_3_ according to: *M*_n,NMR_ = {*M*_NB_ × Int.(δ2.79 + 2.44)} + {*M*_CDT_ × Int.(δ5.41)/3} + M_CTA_.

The average molar mass (*M*_n,SEC_) and dispersity (*Ð*_M_ = *M*_w_/*M*_n_) values of the freshly prepared polymer samples (at most within one week unless otherwise stated) were determined by size exclusion chromatography (SEC) in THF at 30 °C (flow rate = 1.0 mL·min^−1^) on a Polymer Laboratories PL50 apparatus that was equipped with a refractive index detector and a set of two ResiPore PLgel 3 µm MIXED-E 300 × 7.5 mm columns. The polymer samples were dissolved in THF (2 mg·mL^−1^). All elution curves were calibrated with 12 monodisperse polystyrene standards (*M*_n_ range = 580–380,000 g·mol^−1^). *M*_n,SEC_ values of polymers were uncorrected for their possible difference in hydrodynamic volume in THF versus polystyrene. The SEC traces of the polymers all exhibited a monomodal and symmetric peak.

ASAP (Atmospheric Solid Analysis Probe) high-resolution mass spectrometry (HRMS) data were recorded at the CRMPO-Scanmat (Rennes, France) with a Bruker MicrOTOF-Q II mass spectrometer that was equipped with an APCI (Atmospheric Pressure Chemical Ionisation) source in positive mode by direct introduction at 370 °C.

MALDI-ToF mass spectra of polymers were recorded at the CESAMO (Bordeaux, France) on a Voyager mass spectrometer (Applied Biosystems) that was equipped with a pulsed N_2_ laser source (337 nm, 4 ns pulse width) and a time-delayed extracted ion source. Spectra were recorded in the positive-ion mode using the reflectron mode and with an accelerating voltage of 20 kV. A freshly prepared solution of the polymer sample in THF (HPLC grade, 10 mg·mL^−1^) and a saturated solution of *trans*-2-[3-(4-*tert*-butylphenyl)-2-methyl-2-propenylidene]-malononitrile (10 mg, DCTB) in THF (1 mL, HPLC grade) were prepared. A MeOH solution of the cationizing agent (NaI, 10 mg·mL^−1^; Na^+^ ions interact with the polymer’s heteroatoms enabling the observation of DF PCOE) was also prepared. The solutions were combined in a 10:1:1 *v/v/v* of matrix-to-sample-to-cationizing agent. The resulting solution (1–2 μL) was deposited onto the sample target and vacuum-dried.

FTIR spectra of the polymers were acquired (16 scans) with a resolution of 4 cm^−1^ on a IRAffinity-1 (Shimadzu, Champs sur Marne, France) that was equipped with an ATR module.

Differential scanning calorimetry (DSC) analyses were performed with a DSC 131 apparatus (Setaram, Caluire-et-Cuire, France) calibrated with indium, at a rate of 10 °C·min^−1^, under a continuous flow of helium (25 mL·min^−1^), using aluminum capsules. The thermograms were recorded according to the following cycles: −70 to 120 °C at 10 °C·min^−1^; 120 to −70 °C at 10 °C·min^−1^; −70 for 5 min; −70 °C to 120 °C at 10 °C·min^−1^; 120 °C to −70 °C at 10 °C·min^−1^.

Apparent viscosity was measured with an ARES **G2** viscosimeter that was equipped with a plate-plate geometry, at a speed gradient of 0.01 s^−1^ over a shear rate range from 0.01 to 100·s^−1^. At each imposed shear rate, the apparent viscosity was determined in the steady state regime. The temperature was fixed at 23 ± 0.3 °C. For each sample, the viscosimetric test duration was 5 min.

### 2.3. Bis((3-methyloxetan-3-yl)methyl) Fumarate (CTA ***2***)

A three-neck flask (25 mL) was charged with distilled diethyl fumarate (***i***, 0.43 g, 2.5 mmol), (3-methyloxetan-3-yl)methanol (3.65 g, 24.98 mmol), and lipase acrylic resin from *Candida Antarctica* Lipase B (CALB, >5000 U/g, 0.28 g, Q = 570,000 U/mol). The reaction mixture was heated (without stirring) at 60 °C for 7 days (Scheme 2c). The solution was filtrated, and the filtrate was purified by distillation under vacuum (116 °C/12 mmHg) to give CTA **2** as colorless crystals at room temperature (0.70 g, 99%). Crystals suitable for X-ray diffraction studies were selected from this batch (Figure S1). ^1^H NMR (500 MHz, DMSO-*d*_6_, 25 °C): δ (ppm) 6.79 (s, 2H, C*H*C(O)OCH_2_C(CH_3_)CH_2_O), 4.42 (d, *J* = 6.0 Hz, 4H, CHC(O)OCH_2_C(CH_3_)C*H_2_*O), 4.27 (d, *J* = 6.0 Hz, 4H, CHC(O)OCH_2_C(CH_3_)C*H_2_*O), 4.26 (s, 4H, CHCC(O)OC*H_2_*C(CH_3_)CH_2_O), 1.29 (s, 6H, CHC(O)OCH_2_C(C*H_3_*)CH_2_O (Figure S2). ^13^C{^1^H} NMR (125 MHz, DMSO-*d*_6_, 25 °C): δ (ppm) 20.9 (CHC(O)OCH_2_C(*C*H_3_)CH_2_O), 40.8 (CHC(O)OCH_2_*C*(CH_3_)CH_2_O), 66.3 (CHC(O)O*C*H_2_C(CCH_3_)CH_2_O), 78.6 (CHC(O)OCH_2_C(CH_3_)*C*H_2_O), 133.2 (*C*HC(O)OCH_2_C(CH_3_)CH_2_O), 164.3 (C*C*(O)OCH_2_C(CH_3_)CH_2_O) (Figure S3). HRMS (ESI): calcd for C_14_H_20_O_6_Na [M + Na]^+^ theoretical: 307.1157; found: 307.1156 (0 ppm).

### 2.4. Bis((3-ethyloxetan-3-yl)methyl) (E)-Hex-3-enedioate (CTA ***3***)

A solution of (*E*)-hex-3-enedioic acid (3.00 g, 0.021 mol) and *p*-toluenesulfonic acid (0.20 g, 0.001 mol) in ethanol (9.58 g, 0.208 mol) was heated at 90 °C for 72 h. The reaction mixture was then allowed to warm to room temperature for 1 h. The filtrate was washed with brine (2 × 30 mL), dried, and concentrated. The recovered residue was purified by distillation under vacuum (110 °C/12 mmHg) to give diethyl fumarate (***ii***) as a colorless oil (4.15 g, 99%). ^1^H NMR (500 MHz, DMSO-*d*_6_, 25 °C): δ (ppm) 1.33 (t, *J* = 7 Hz, 6H, C*H_3_*CH_2_OC(O)CH_2_CH), 3.16 (m, 4H,CH_3_CH_2_OC(O)C*H_2_*CH), 4.21 (q, *J* = 7 Hz, 4H, CH_3_C*H_2_*OC(O)CH_2_CH), 5.77 (m, 2H, CH_3_CH_2_OC(O)CH_2_C*H*). A three-neck flask (25 mL) was charged with (***ii***) (2.00 g, 10 mmol), (3-ethyloxetan-3-yl)methanol (9.27 g, 80 mmol), and lipase acrylic resin from *Candida Antarctica* Lipase B (CALB, >5000 U/g, 1.13 g, Q = 570,000 U/mol). The reaction mixture was heated at 60 °C for 7 days. The mixture was filtrated, and the filtrate was purified by distillation under vacuum (110 °C/12 mmHg) to give CTA **3** as a colorless oil (3.50 g, 99%). ^1^H NMR (500 MHz, DMSO-*d*_6_, 25 °C): δ (ppm) 5.65 (s, 2H, C*H*CH_2_C(O)OCH_2_C(CH_2_CH_3_)CH_2_O), 4.33 (d, *J* = 6.0 Hz, 4H, CHCH_2_C(O)OCH_2_C(CH_2_CH_3_)C*H_2_*O), 4.26 (d, *J* = 6.0 Hz, 4H, CHCH_2_C(O)OCH_2_C(CH_2_CH_3_)C*H_2_*O), 4.18 (s, 4H, CHCH_2_C(O)OC*H_2_*C(CH_2_CH_3_)CH_2_O), 3.14 (d, *J* = 5 Hz, 4H, CHC*H_2_*C(O)OCH_2_C(CH_2_CH_3_)CH_2_O), 1.65 (d, *J* = 7.5 Hz, 2H, CHCH_2_C(O)OCH_2_C(C*H_2_*CH_3_)CH_2_O), 0.85 (t, *J* = 7.5 Hz, 6H, CHCH_2_C(O)OCH_2_C(CH_2_C*H_3_*)CH_2_O) (Figure S4). ^13^C{^1^H} NMR (125 MHz, DMSO-*d*_6_, 25 °C): δ (ppm) 8.0 (CHCH_2_C(O)OCH_2_C(CH_2_*C*H_3_)CH_2_O), 26.1 (CHCH_2_C(O)OCH_2_C(*C*H_2_CH_3_)CH_2_O), 37.0 (CH*C*H_2_C(O)OCH_2_C(CH_2_CH_3_)CH_2_O), 42.1 (CHCH_2_C(O)OCH_2_*C*(CH_2_CH_3_)CH_2_O), 66.1 (CHCH_2_C(O)O*C*H_2_C(CH_2_CH_3_)CH_2_O), 76.5 (CHCH_2_C(O)OCH_2_C(CH_2_CH_3_)*C*H_2_O), 125.9 (*C*HCH_2_C(O)OCH_2_C(CH_2_CH_3_)CH_2_O), 171.2 (CHCH_2_*C*(O)OCH_2_C(CH_2_CH_3_)CH_2_O) (Figure S5). HRMS (ESI): calcd for C_18_H_28_O_6_Na [M + Na]^+^ theoretical: 363.1784; found: 363.1787 (1 ppm).

### 2.5. General Procedure for the Polymerization of COE

**Figure 2:**
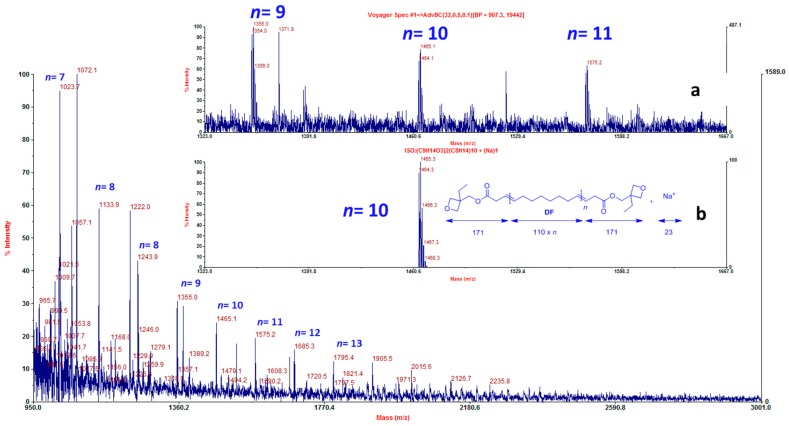
MALDI-ToF mass spectrum (DCTB matrix, Na ionizing salt) of an α,ω-dioxetane telechelic PCOE sample that was prepared by ROMP/CM of COE in the presence of **G2**/CTA **3** (Table 1, entry 4); see top zoomed region and the corresponding bottom simulation for *n* = 10.

All polymerizations were performed according to the following typical procedure (Table 1, entry 1). The only differences lie in the nature of the solvent, the catalyst loading, the CTA, and the CTA’s initial concentration ([CTA]_0_). Under an argon atmosphere, a Schlenk flask that was equipped with a magnetic stir bar was charged sequentially with dry CH_2_Cl_2_ (5.0 mL), COE (1.53 mL, 1.29 g, 11.7 mmol), and CTA **2** (66.5 mg, 0.234 mmol). The initial concentration of COE was kept at 1.8 mol·L^−1^. The resulting solution was heated at 40 °C, and the polymerization was started upon addition, via a cannula, of a freshly prepared CH_2_Cl_2_ solution (2.0 mL) of **G2** (9.9 mg, 11.7 μmol). The reaction mixture turned highly viscous within 2 min. The viscosity then slowly decreased over the following 10 min. After the desired reaction time (typically 24 h; reaction time was not necessarily optimized), volatiles were removed under vacuum. The polymer was recovered upon precipitation in methanol (50 mL) (thereby allowing for removal of the catalyst), filtration, and drying at 25 °C under vacuum. All polymers were recovered as white powders, which are readily soluble in CHCl_3_ and THF, and insoluble in MeOH (Table 1). All experiments were at least duplicated. The isolated polymers were characterized by ^1^H NMR spectroscopy, MALDI-ToF MS, and DSC analyses (Figures 1 and 2, Table 5).

### 2.6. General Procedure for the Copolymerization of Cyclic Olefins

**Figure 3:**
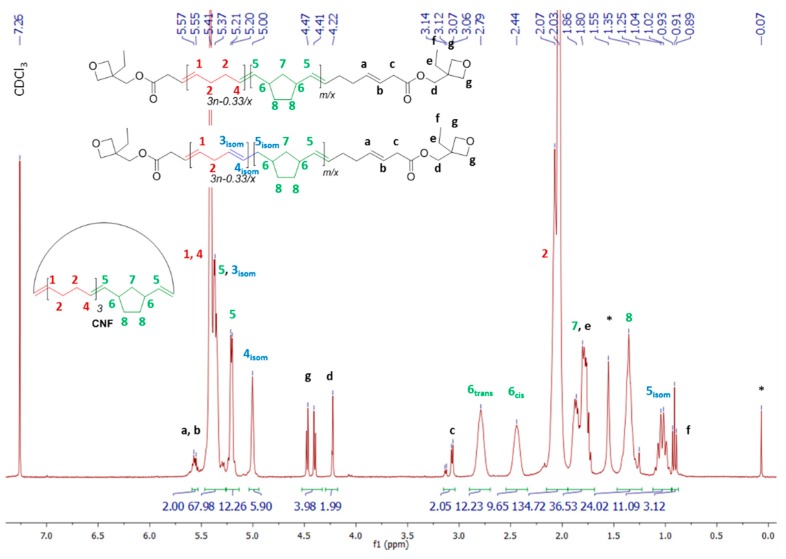
^1^H NMR spectrum (500 MHz, CDCl_3_, 25 °C) of an α,ω-dioxetane telechelic P(NB-*co*-CDT) copolymer sample that was prepared by ROMP/CM of an equimolar mixture of NB/CDT in the presence of **G2**/CTA **3** in CH_2_Cl_2_ (Table 3, entry 8) (* δ (ppm) 1.55 residual H_2_O, 0.07 residual grease).

**Figure 4:**
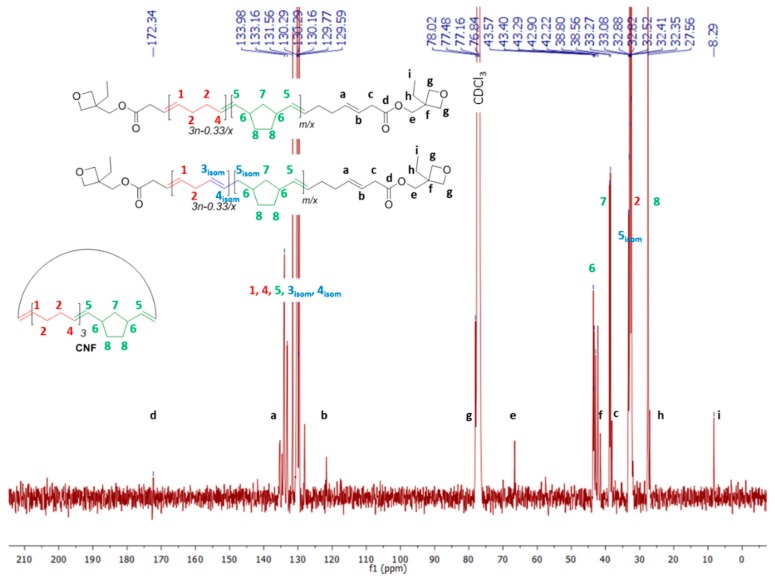
^13^C{^1^H} NMR spectrum (125 MHz, CDCl_3_, 25 °C) of an α,ω-oxetane telechelic P(NB-*co*-CDT) copolymer sample that was prepared by ROMP/CM of an equimolar mixture of NB/CDT in the presence of **G2**/CTA **3** in CH_2_Cl_2_ (Table 3, entry 8).

All copolymerizations were performed according to the following typical procedure that is described thereafter for NB and CDT. Typically, under an argon atmosphere, a Schlenk flask that was equipped with a magnetic stir bar was charged sequentially with 1,2-dichloroethane (10.0 mL), NB (0.93 g, 9.87 mmol), CDT (1.80 mL, 1.60 g, 9.87 mmol), and CTA **3** (168 mg, 0.49 mmol) (Table 3, entry 8). The resulting solution was heated at 60 °C, and the polymerization was started upon addition, via a cannula, of a dry, freshly prepared CH_2_Cl_2_ solution (4.0 mL) of **G2** (5.9 mg, 7.01 μmol). Note that reactions performed in CH_2_Cl_2_ or THF were run at 40 or 60 °C, respectively. The reaction mixture turned highly viscous within 2 min. The viscosity then visually slowly decreased over the following 10 min. After the desired reaction time (typically 24 h; reaction time was not necessarily optimized), volatiles were removed under vacuum. The isolated copolymers were characterized by ^1^H, ^13^C{^1^H} NMR spectroscopy, DSC, and viscosimetric analyses (Tables 2, 3, 5, and 6; Figures 3, 4 and S6–S11).

### 2.7. Chemical Modification of α,ω-Diepoxide Telechelic P(NB-co-CDT) into α,ω-Bis(dithiocarbonate) Telechelic P(NB-co-CDT)

**Figure 5:**
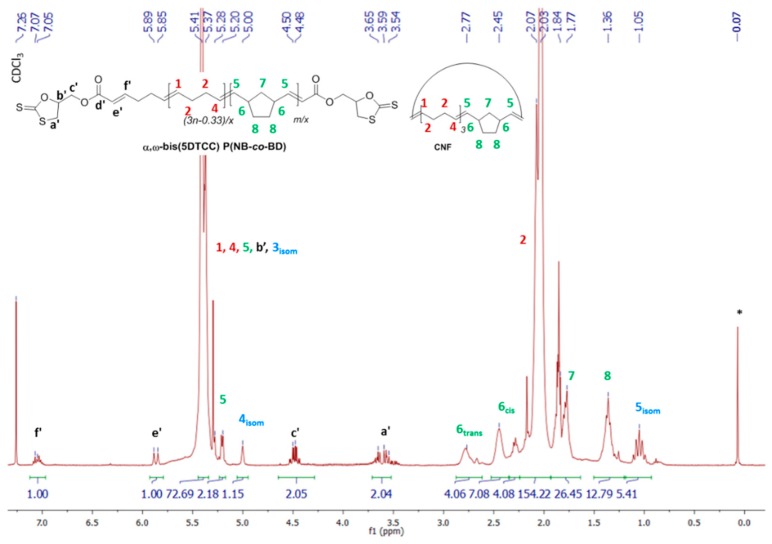
^1^H NMR spectrum (500 MHz, CDCl_3_, 25 °C) of an α,ω-bis(dithiocarbonate) P(NB-*co*-CDT) (Table 4, entry 1) copolymer sample that was prepared upon dithiocarbonatation of an α,ω-bis(dioxetane) P(NB-*co*-CDT) copolymer with CS_2_/LiBr (Table 3, entry 5).

**Figure 6:**
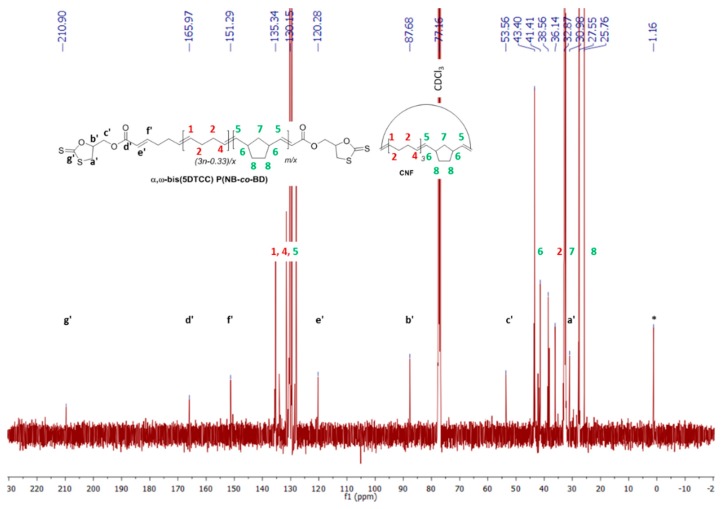
^13^C{^1^H} NMR spectrum (125 MHz, CDCl_3_, 25 °C) of an α,ω-bis(dithiocarbonate) P(NB-*co*-CDT) (Table 4, entry 1) copolymer sample that was prepared upon dithiocarbonatation of an α,ω-bis(dioxetane) P(NB-*co*-CDT) copolymer with CS_2_/LiBr (Table 3, entry 5).

α,ω-Diepoxide telechelic P(NB-*co*-CDT)s were quantitatively chemically modified into their analogous copolymers end-capped with dithiocarbonate groups according to our previously established procedure [39]. The reaction was performed in THF for 17 h, according to a modified literature procedure [43], upon optimization of the initial stoichiometry, thus using α,ω-diepoxide P(NB-*co*-CDT) (1 equiv.), LiBr (2.0 equiv.), and CS_2_ (2.2 equiv.). The isolated copolymers were characterized by ^1^H, ^13^C{^1^H} NMR and FTIR spectroscopy, DSC, and viscosimetric analyses (Figures 5 and 6; Tables 4–6 and S1, Figures S12–S13).

### 2.8. Quantification of Cyclic Nonfunctional (CNF) Copolymers within the Recovered Chemically Modified P(NB-co-CDT) Crude Copolymers

CNF copolyolefins were separated from the crude α,ω-bis(dithiocarbonate) P(NB-*co*-CDT) copolymers by eluting a mixture that was issued from the crude recovered copolymers with a multifunctional amine (Lupasol FG^®^) (1.5 equiv NH_2_ versus dithiocarboante) in CH_2_Cl_2_ (50 mL), through a silica column chromatography, using pentane as eluent. The recovered CNF polymers were then weighted and characterized by ^1^H NMR spectroscopy (Figure S13).

## 3. Results and Discussion

### 3.1. Synthesis of CTAs ***1**–**3***

**Scheme 1:**
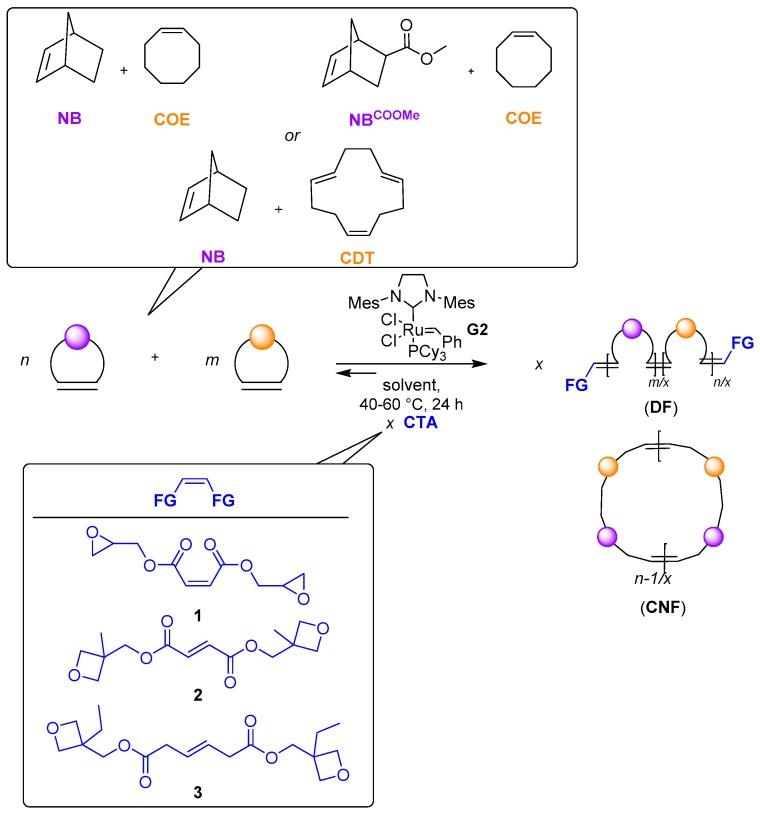
Tandem ROMP/CM of cycloolefins (NB/COE, NB/CDT, or NB^COOMe^/COE; equimolar mixtures) catalyzed by Grubbs’ second generation catalyst **G2** in the presence of a difunctional alkene CTA **1**, **2**, or **3**, showing the possible copolymers (FG: epoxide or oxetane functional group; DF: α,ω-difunctional, CNF: cyclic nonfunctional).

Both oxetane- and epoxide-functional CTAs have been developed in order to access the corresponding α,ω-difunctional coPOs (Scheme 1). While the bis(oxiran-2-ylmethyl) maleate CTA **1** was prepared according to the previously reported procedure [44], the two oxetane functional CTAs were prepared by sequential transesterification reactions. CTA **3**, including, as compared to CTA **2**, an additional methylene unit between the ethylenyl double bond and the ester function, was next selected in order to evaluate the impact of the delocalization of C=C electrons onto the cross-metathesis reaction [45]. Also, the oxetane-based CTAs may, upon ultimate chemical modification of the oxetane end-capped polymers through (thio)carboxylation, lead to the corresponding six-membered ring (thio)carbonate end-functional polymers. Indeed, such telechelic polymers may be useful for the subsequent preparation of, for instance, non-isocyanate polyurethanes [46].

**Scheme 2:**
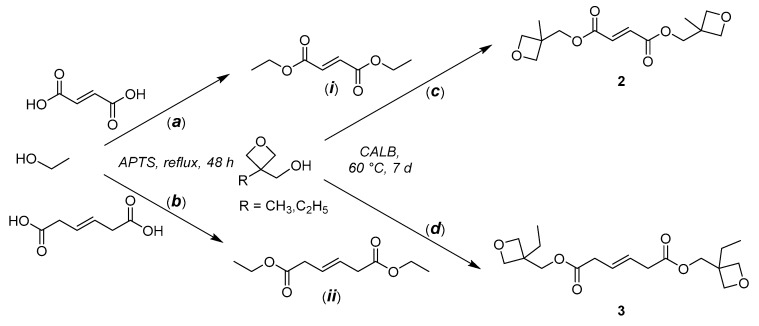
Synthesis of the oxetane-functional CTAs **2** and **3**.

First, diethyl fumarate (***i***) reacted with (3-methyloxetane-3-yl)methanol in the presence of *Candida Antarctica* Lipase B (CALB) immobilized onto an acrylic resin to afford bis(oxetane-2-ylmethyl) maleate CTA **2** (Scheme 2c) [47,48]. Diethyl fumarate (***i***) was previously obtained from the esterification of fumaric acid and ethanol (Scheme 2a). Similarly, reaction of (*E*)-hex-3-ene dioic acid with ethanol formed compound (***ii***) (Scheme 2b), which upon transesterification with (3-methyloxetane-3-yl)ethanol generated bis(oxetane-2-ylmethyl) (*E*)-hex-3-enedioate CTA **3** (Scheme 2d). Both CTAs **2**–**3** were isolated in quantitative yield and characterized by ^1^H, ^13^C{^1^H} NMR, single-crystal X-ray diffraction (for **2**), and HRMS analyses (Figures S1–S5).

### 3.2. Metathesis of COE in the Presence of CTAs ***1**–**3***

The use of the symmetric epoxide CTA **1** for the preparation of the corresponding epoxide-telechelic PCOE homopolymer by ROMP/CM [37,38] of cyclooctene (COE) using **G2** was already successfully established [39]. The efficiency of the ROMP/CM of COE using **G2** in the presence of the new oxetane symmetric CTAs **2**–**3** to provide the corresponding oxetane-telechelic PCOEs was, thus, first evaluated, prior to the synthesis of epoxide- and oxetane-end-capped coPOs (vide infra).

The tandem ROMP/CM of COE catalyzed by **G2** in the presence of oxetane CTAs **2** and **3** was investigated in CH_2_Cl_2_ at 40 °C over 24 h (Table 1). Although the COE was always fully consumed, the conversion of CTA **2** remained sluggish and incomplete (≤20%), even decreasing with larger amounts of CTA (Table 1, entries 1–3). On the other hand, the metathesis reaction using CTA **3** showed the complete conversion of both COE and CTA; in this case, the molar mass values as determined by a ^1^H NMR analysis (*M*_n,NMR_) fairly matched the data that were calculated (*M*_n,theo_) on the basis of the formation of only DF PCOEs (*M*_n,theo_) (i.e., without taking into account any CNF nor any nonfunctional PCOE; refer to the Experimental Section) (Table 1, entries 4, 5). The observed difference between the molar mass values as measured from the SEC analysis (*M*_n,SEC_) and *M*_n,NMR_ or *M*_n,theo_ most likely reflects the difference in the hydrodynamic radius between the PCOEs and the polystyrene standards that were used for the calibration of the SEC apparatus, as previously observed [34,35,36,37,38,39,40,41,42]. Nevertheless, the *M*_n,SEC_ values proportionaly increased with larger amounts of monomer consumed. The additional methylene group in between the C=C double bond and the ester group in CTA **3** versus CTA **2** thus significantly improved the efficiency of the tandem metathesis reaction, as similarly reported with alike epoxyde or azlactone symmetric CTAs [39,42].

The PCOE thus formed displayed the expected oxetane chain end-capping groups as evidenced by NMR spectroscopy and mass spectrometry analyses. The ^1^H NMR spectrum showed the characteristic signals of COE repeating units (H^1^–H^3^) along with the typical C*H_2_*O methylene groups (δ (ppm) 4.44 and 4.23; Figure 1) of the oxetane moiety. Correspondingly, the MALDI-ToF mass spectrum (MS) clearly evidenced the α,ω-dioxetane PCOE as the major population observed with a repeating unit of 110 g·mol^−1^, with e.g., *m/z*_experimental_ = 1465.1 g·mol^−1^ versus *m/z*_simulated_ = 1465.3 g·mol^−1^ for *n* = 10 (Figure 2). The oxetane-based CTAs were, thus, revealed to be stable in the presence of **G2** and suitably enabled the preparation of α,ω-dioxetane PCOE, thus foreseeing the successful preparation of coPOs, more specifically using CTA **3**.

### 3.3. Ring-Opening Metathesis Copolymerization of Cyclic Olefins in the Presence of CTAs ***1*** or ***3***

The two most efficient CTAs, namely **1** and **3**, were selected for the copolymerization studies. Commercially available monomers where herein considered towards the synthesis of telechelic copolymers, namely COE, NB, and the related NB featuring a methyl ester substituent, NB^COOMe^. The molar ratio of comonomers was fixed at 50:50, based on previous investigations that revealed such resulting copolymers with equal composition in each comonomer to display a lower viscosity, thereby being more attractive towards the elaboration of low viscosity liquid polymer materials that are aimed at adhesive or coating applications [38,39,40,41,42].

### 3.4. α,ω-Diepoxide Telechelic P(COE-co-NB) and P(COE-co-NB^COOMe^) Copolymers

The ROMP of COE and NB or NB^COOMe^ with **G2** in the presence of CTA **1** (the most easily accessible CTA (one-step; high yield)) was similarly performed at 40 °C for 24 h in CH_2_Cl_2_ (Scheme 1). An average conversion in CTA **1** of 75% was reached at lower COE/NB loadings (typically 500 mol. equiv. each versus **G2**), while increasing the initial content in both comonomers resulted in a lower CM efficiency (Table 2, entries 1–4). In comparison, the homopolymerization of COE under similar operating conditions was as effective [39]. On the other hand, the copolymerization of NB with CDT under similar operating conditions was revealed to be ineffective (vide infra, Table 3, entries 1–2). This suggested the beneficial presence of COE for its successful copolymerization with NB. Similarly, the COE/NB^COOMe^ copolymerization proceeded fairly with a ca. 45% conversion of CTA **1** with a loading of comonomers as high as 1000 mol. equiv. (Table 2, entries 5–6). The experimental molar mass values determined by NMR matched well the calculated values, while the dispersity remained within the common range for coPOs (*Ð*_M_ <1.5), thus highlighting a fairly controlled polymerization. The NMR characterizations of the copolymers demonstrated the successful formation of the corresponding random copolymers end-capped at both termini by the epoxide moiety, in agreement with data in the literature on homoPCOEs [39], as illustrated in Figures S6–S7.

### 3.5. α,ω-Diepoxide and -Dioxetane Telechelic P(NB-co-CDT) Copolymers

The one-pot simultaneous copolymerization of NB and CDT catalyzed by **G2** or the Hoveyda–Grubbs catalyst **HG2** in the presence of CTA **1** or **3** was performed at 40 or 60 °C for 24 h in CH_2_Cl_2_ or (CH_2_Cl)_2_ (Table 3, Scheme 1).

As aforementioned, attempts to copolymerize NB and CDT (50:50 mol/mol) in the presence of the catalytic system **G2**/CTA **1** in CH_2_Cl_2_ at 40 °C failed, as evidenced by a ^1^H NMR analysis that showed the presence of unreacted CTA **1** (Table 3, entries 1–2, Figure S8). Replacing **G2** by the Hoveyda–Grubbs catalyst **HG2** enabled us to reach a good CM efficiency (up to ca. 80% CTA **1** consumption) when the reaction temperature was raised to 60 °C and using THF as the reaction medium (Table 3, entries 3–7). Well-defined copolymers with controlled molar mass values (i.e., *M*_n,NMR_ values close to the anticipated ones *M*_n,theo_, fairly narrow dispersity values) were isolated after purification by dialysis in THF (so as to remove unreacted CTA **1**). Both the ^1^H and ^13^C NMR spectra supported the formation of NB/CDT copolymers end-capped with glycidyl moieties (Figures S9 and S10). Indeed, the spectra exhibited the main chain olefinic (NB and CDT/butadiene) hydrogens along with the typical signals of the glycidyl α,β-unsaturated carboxylate end-groups, similarly to the diepoxide telechelic PCOE homopolymers spectra [39], thereby demonstrating the formation of DF and possibly CNF P(NB-*co*-CDT) copolymers.

In contrast, the similar ROMP/CM of NB and CDT (50:50) with CTA **3** mediated by **G2** in CH_2_Cl_2_ was effective (Table 3, entries 8–9). The CM proceeded to completion (quantitative CTA **3** conversion) affording P(NB-*co*-CDT) copolymers with the expected molar mass value as determined by a ^1^H NMR analysis, yet with a somewhat large dispersity. Characterization of the copolymers by ^1^H and ^13^C{^1^H} NMR spectroscopy supported the formation of α,ω-dioxetane telechelic P(NB-*co*-CDT) copolymers (Figure 3 and Figure 4). The typical ^1^H spectrum that is depicted in Figure 3 showed both the CDT/butadiene (δ (ppm) 5.41 (H^1,4^), 2.03 (H^2^); in red) and NB (δ (ppm) 5.41 (H^5^), 1.86 (H^7^), 1.25 (H^8^); in green) signature; two signals for the NB repeating units (δ (ppm) 2.79 (H^6trans^), 2.44 (H^6cis^); in green) were clearly observed, indicating both the *cis* and *trans* configurations of the C=C bond of the NB/butadiene junction (C^4^=C^5^). In addition, these double bonds were found to have migrated within the CDT/butadiene polymer’s backbone (δ (ppm) 5.39 (H^3isom^), 5.00 (H^4isom^), 1.02 (H^5isom^); in blue), evidencing side isomerization. Finally, typical oxetane signals (δ (ppm) 5.56 (H^a,b^), 4.47–4.41 (H^g^), 4.22 (H^d^), 3.14–3.06 (H^c^), 1.80 (H^e^), 0.91 (H^f^); in black) were unambiguously identified. Remarkably, the oxetane end-groups are always linked to a CDT/butadiene unit, and not to NB, as also observed in the presence of CTA **1** (this was, however, not the case with alike azlactone end-capped copolyolefins [42]. This linkage mode was further evidenced by a 2D COSY ^1^H–^1^H NMR analysis that displayed the correlation peak between the oxetane hydrogens H^a,b^ and H^c^ with the CDT/butadiene hydrogen H^2^, while no cross peak was observed between H^a,b^ and H^6^ hydrogens, evidencing the absence of an oxetane/NB direct bond (Figure S11). Correspondingly, the ^13^C{^1^H} NMR spectrum distinctively showed the typical NB (C^6^, C^7^, C^8^; in green) and CDT/butadiene (C^1^, C^2^, C^4^; in red) repeating units signals, along with those of the terminal oxetane function (C^a^–C^i^; in black) (Figure 4). However, the ^13^C signals of the C=C isomerized bond (C^3isom^, C^4isom^, C^5isom^; in blue), and those of the C^6^ carbon in the *cis* or *trans* configuration of the C=C bond, were not resolved.

### 3.6. Post-Polymerization Chemical Modification of α,ω-Di(epoxide) Telechelic P(NB-co-CDT) into α,ω-Dithiocarbonate Telechelic P(NB-co-CDT)

**Scheme 3:**
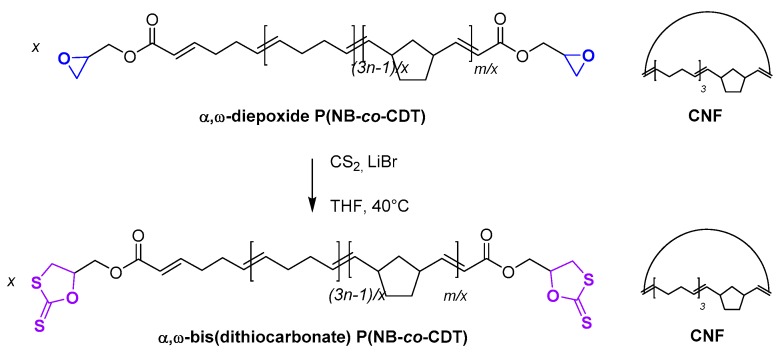
Chemical modification of α,ω-diepoxide telechelic P(NB-*co*-CDT) copolymers into α,ω-bis(dithiocarbonate) P(NB-*co*-CDT) analogues.

α,ω-Diepoxide telechelic P(NB-*co*-CDT) copolymers were next chemically modified into the analogous copolymers featuring dithiocarbonate termini according to our previously established procedure [39]. Indeed, we have previously demonstrated that dithiocarbonate-based CTAs are not chemically compatible with the **G2** catalyst, thus precluding the direct preparation of dithiocarbonate end-functional coPOs by the tandem ROMP/CM approach [39]. The reaction with CS_2_/LiBr at 40 °C was performed in THF to improve the homogeneity of the reaction medium (α,ω-diepoxide telechelic P(NB-*co*-CDT) copolymers are of low viscosity, yet still viscous) (Scheme 3) (Table 4 and Table 6). As anticipated, the molar mass values *M*_n,NMR_ and *M*_n,sec_ were not significantly impacted by the chemical modification of the epoxide chain-end groups (Table 4) [39].

^1^H and ^13^C{^1^H} NMR and FTIR analyses of the α,ω-bis(dithiocarbonate) P(NB-*co*-CDT) copolymers supported the successful formation of dithiocarbonate chain ends. The NMR signals corresponding to the NB and CDT repeating units were clearly observed in the spectra of the prepolymer (Figure 5 and Table 6 versus Figures S9 and S10; Table S1). In addition, the typical signals of the dithiocarbonate moiety corresponding to the C*H_2_*–S (H^a′^, δ (ppm) 3.59) and C*H_2_*–OC(O) (H^c′^, δ (ppm) 4.49), the former one being clearly deshielded relative to the related signal in the prepolymer (H^a^, δ (ppm) 2.67), were unambiguously identified. Note that the dithiocarbonate methine signal (H^b′^, δ (ppm) 5.37) could not be used to evidence the successful thiocarbonatation reaction as it overlaps with the main chain signals. The dithiocarboante moiety was clearly evidenced in the ^13^C{^1^H} NMR spectrum by the C=S signal (C^g′^ δ (ppm) 210.9, Figure 6). Note that the diagnostic signal of CS_2_ (δ 193.1 ppm) was not observed in the spectrum, within the NMR detection limits. Finally, the ATR FTIR spectrum also clearly displays the dithiocarboante C=S and C=O absorption bands at ν = 1192 and 1520 cm^−1^, respectively (Figure S12).

Evaluation of the amount of cyclic nonfunctional polymer (CNF) within the recovered chemically modified P(NB-*co*-CDT) crude copolymers was performed upon eluting with pentane a CH_2_Cl_2_ mixture of the latter samples with a multifunctional amine (Lupasol FG^®^; 1.5 equiv. NH_2_ versus dithiocarbonate) through a silica column. Reaction of the dithiocarbonate groups with the amine resulted in cross-linking of the α,ω-bis(dithiocarbonate) P(NB-*co*-CDT), while eluting the apolar CNF copolymer. A ^1^H NMR analysis of the eluted sample did not show the signals of the dithiocarbonate moiety (H^a′^–H^c′^ in Figure 5) or related functional groups obtained upon reaction with amines, but displayed only signals from NB and CDT repeating units, in agreement with the elution of only CNF copolymers (Figure S13). Weighting of the thus recovered CNF gave an average weight content of <ca. 10wt%. This value falls within the range of CNF contents previously reported during the alike ROMP/CM metathesis preparation of telechelic POs [38,39,40,41,42]. The alternative quantification of the CNF by ^1^H NMR spectroscopy using an internal standard gave the same amounts of CNF.

### 3.7. Thermal Properties of the Polymers

The nature of the end-capping group of telechelic PCOEs as well as of P(NB-*co*-CDT) copolymers, either epoxide, oxetane, or dithiocarbonate, was found not to impact the thermal transition temperatures (Table 5). PCOEs terminated with epoxide or oxetane did not display a clear glass transition, similarly to other reported telechelic PCOEs, such as those functionalized with glycidyl alkenoate (*M*_n,NMR_ = 6500 g·mol^‒1^, *T*_g_ = not observed, *T*_m_ = 57 °C, *T*_c_ = 45 °C) [39], carboxylate (*M*_n,NMR_ = 5000 g·mol^‒1^, *T*_g_ = not observed, *T*_m_ = 62 °C, *T*_c_ = 51 °C) [15], dithiocarbonate (*M*_n,NMR_ = 6500 g·mol^‒1^, *T*_g_ = not observed, *T*_m_ = 56 °C, *T*_c_ = 43 °C) [39], or azlactone (*M*_n,NMR_ = 5900 g·mol^‒1^, *T*_g_ = not observed, *T*_m_ = 56 °C, *T*_c_ = 47 °C) [42] α,ω-end-functional PCOEs. Also, the *T*_m_ value of PCOEs is known to inform on the *cis*/*trans* configuration of the C=C bonds along the PO backbone. *T*_m_ values of PCOEs ranging from 10–54 °C are indicative of 99%–20% of *cis* C=C bonds, respectively [49]. Correlation of the experimentally observed *T*_m_ values of the telechelic PCOEs that are gathered in Table 5 thus suggested ca. 20% of *cis* C=C bonds along the polymer’s backbone. All of the P(NB-*co*-CDT)s end-capped with epoxide, oxetane, or dithiocarbonate moieties showed a very similar thermal signature, similar to the one that was reported for azlactone end-capped PCOEs, although a glass transition was not observed in the latter copolymers (*M*_n,NMR_ = 6900 g·mol^‒1^, *T*_g_ = not observed, *T*_m_ = 7 °C, *T*_c_ = −18 °C) [42]. It is noteworthy that PCOE (*M*_n,NMR_ = 2900 g·mol^‒1^, *T*_g_ = −78 °C, *T*_m_ = 52 °C, *T*_c_ = 45 °C) and P(NB-*co*-CDT) (*M*_n,NMR_ = 2900 g·mol^‒1^, *T*_g_ = not observed, *T*_m_ = 19 °C, *T*_c_ = −3 °C) α,ω-end-functionalized with trimethoxysilyl groups exhibited a thermal profile that was distinct from these telechelic coPOs [40].

### 3.8. Viscosity of the Copolymers

The viscosity properties of the epoxide, oxetane, and dithiocarbonate telechelic copolymers were investigated at ambient temperature in simple shear flows (Table 6). Epoxide telechelic P(COE-*co*-NB) and P(COE-*co*-NB^COOMe^) copolymers showed a rather high, and similar, viscosity regardless of the presence of the methyl ester substituent on the NB units (Table 6, entries 1–2). This suggested that the functional NB^COOMe^ did not alter the linearity of the P(COE-*co*-NB) copolymer (a longer alkyl group—e.g., hexyle—on the ester may affect the viscosity more significantly), and that COE most likely enabled an increase in the viscosity of NB segments. In comparison, the viscosity of P(NB-*co*-CDT) copolymers showed, regardless of the terminal epoxy, oxetane, or dithiocarbonate functionality, a much lower viscosity, most likely imparted by the CDT segments. The latter copolymers also exhibited the same viscosity as the related azlactone telechelic P(NB-*co*-CDT) copolymers [42]. All copolymers displayed a shear thinning rheological behavior.

## 4. Conclusions

Both α,ω-diepoxide and -dioxetane telechelic (co)POs have been straightforwardly and selectively prepared from the tandem ROMP/CM polymerization of COE, of COE with NB or NB^COOMe^, or of CDT with NB, catalyzed by **G2** in the presence of the symmetrical epoxide or oxetane functional alkene CTA, respectively. The CM was found to be more effective for CTA **3** > **1** >> **2**; this further highlights the key importance of the central C=C moiety in these chain-transfer agents, where both electronic and steric considerations must be taken into account. Well-defined α,ω-di(epoxide or oxetane) telechelic PCOEs, P(COE*-co-*NB or -NB^COOMe^)s, and P(NB*-co-*CDT)s were, thus, obtained under mild operating conditions (solvent, 40–60 °C, 24 h). The post-metathesis polymerization quantitative chemical modification (CS_2_/LiBr, 40 °C, THF) of α,ω-di(epoxide) telechelic P(NB-*co*-CDT) successfully afforded a convenient approach to the corresponding α,ω-bis(dithiocarbonate) telechelic P(NB-*co*-CDT). All of the (co)polymers display spectroscopic (NMR, FTIR) and spectrometric (MS) data evidencing the polyolefinic backbone along with the respective chain-end functions. Cross-metathesis of CTAs takes place selectively on COE or CDT segments, and not on NB units. The DSC signature and the low viscosity liquid behavior of the epoxide/oxetane/dithiocarbonate telechelic P(NB-*co*-CDT)s were not influenced by the nature of the termini. Overall, this work establishes the first reported preparation of both α,ω-di(oxetane) and α,ω-bis(dithiocarbonate) telechelic copolyolefins.

## Figures and Tables

**Table 1:** ROMP/CM of COE catalyzed by **G2** in the presence of oxetane CTAs **2** and **3** in CH_2_Cl_2_ at 40 °C over 24 h (Scheme 1) *^a^*.

Entry	CTA	[COE]_0_/[CTA]_0_/[G2]_0_	CTA Conv. *^b^* (%)	*M*_n,theo_*^c^* (g·mol^−1^)	*M*_n,NMR_*^d^* (g·mol^−1^)	*M*_n,SEC_*^e^* (g·mol^−^^1^)	*Ð*_M_*^e^*
1	**2**	1000:20:1	20	27,800	30,300	56,800	1.6
2	**2**	2000:20:1	20	55,300	55,800	95,600	1.5
3	**2**	2000:100:1	<5	>44,300	69,700	127,600	1.6
4	**3**	1000:30:1	100	4000	3700	13,300	1.5
5	**3**	1000:40:1	100	3100	2900	7500	1.5

*^a^* General operating conditions: Catalyst = 5.9 µmol, [COE]_0_ = 1.8 mol·L^−1^; COE and NB conversion = 100% as determined by a ^1^H NMR analysis (refer to the Experimental Section). *^b^* Conversion in CTA as determined by a ^1^H NMR analysis (refer to the Experimental Section). *^c^* Theoretical molar mass value calculated from *M*_n,theo_ = {*M*_COE_ × ([COE]_0_ × Conv._COE_)/([CTA]_0_ × Conv._CTA_)} + *M*_CTA_, with *M*_COE_ = 110 g·mol^−1^, *M*_CTA**2**_ = 284 g·mol^−1^, and *M*_CTA**3**_ = 340 g·mol^−1^, on the basis of the formation of only DF (i.e., without taking into account any CNF PCOEs). *^d^* Experimental molar mass value determined by a ^1^H NMR analysis (refer to the Experimental Section). *^e^* Number-average molar mass (*M*_n,SEC_) and dispersity (*Ð*_M_ = *M*_w_/*M*_n_) values determined by SEC versus polystyrene standards (uncorrected *M*_n_ values) in THF at 30 °C.

**Table 2:** ROMP/CM of COE with NB or NB^COOMe^ (equimolar mixtures) catalyzed by **G2** in the presence of CTA **1** in CH_2_Cl_2_ at 40 °C over 24 h (Scheme 1) *^a^*.

Entry	NB or NB^COOMe^	[NB^(COOMe)^]_0_/[COE]_0_/[CTA 1]_0_/[G2]_0_ *^a^*	CTA Conv. *^b^* (%)	*M_n_*_,theo_*^c^* (g·mol^−1^)	*M*_n,NMR_*^d^* (g·mol^−1^)	*M*_n,SEC_*^e^* (g·mol^−^^1^)	*Ð*_M_*^e^*
1	NB	500:500:20:1	75	7000	9100	20,100	1.5
2	NB	500:500:30:1	70	5100	6600	17,500	1.4
3	NB	500:500:50:1	78	2800	3600	9700	1.3
4	NB	1000:1000:200:1	31	3500	5500	12,400	1.5
5	NB^COOMe^	1000:1000:20:1	56	23,600	22,700	52,400	1.3
6	NB^COOMe^	1000:1000:50:1	40	13,300	23,000	51,700	1.4

^*a*^ General operating conditions: Catalyst = 5.9 µmol, [COE + NB or NB^COOMe^]_0_ = 1.8 mol·L^−1^; COE, NB, and NB^COOMe^ conversion = 100% as determined by a ^1^H NMR analysis (refer to the Experimental Section). ^*b*^ Conversion in CTA as determined by a ^1^H NMR analysis (refer to the Experimental Section). ^*c*^ Theoretical molar mass value calculated from *M*_n,theo_ = {(*M*_NB_ × ([NB]_0_ × Conv._NB_) + *M*_COE_ × ([COE]_0_ × Conv._COE_))/([CTA]_0_ × Conv._CTA_)} + *M*_CTA_, with *M*_COE_ = 110 g·mol^−1^, *M*_NB_ = 94 g·mol^−1^, *M*_NBCOOMe_ = 152 g·mol^−1^, and *M*_CTA**1**_ = 228 g·mol^−1^, on the basis of the formation of only DF (i.e., without taking into account any CNF polymers). ^*d*^ Experimental molar mass value of the copolymer determined by ^1^H NMR analysis (refer to the Experimental Section). ^*e*^ Number-average molar mass (*M*_n,SEC_) and dispersity (*Ð*_*M*_ = *M*_w_/*M*_n_) values determined by SEC versus polystyrene standards (uncorrected *M*_n_ values) in THF at 30 °C.

**Table 3:** ROMP/CM of equimolar mixtures of NB with CDT catalyzed by **G2** or **HG2** in the presence of CTA **1** or **3** in CH_2_Cl_2_ or (CH_2_Cl)_2_ at 40 or 60 °C for 24 h *^a^*.

Entry	CTA	Solvent	Cat.	Temp. (°C)	[NB]_0_/[CDT]_0_/ [CTA]_0_/[Cat.]_0_	CTA Conv. *^b^* (%)	*M_n_*_,theo_*^c^* (g·mol^−1^)	*M*_n,NMR_*^d^* (g·mol^−1^)	*M*_n,SEC_*^e^* (g·mol^−^^1^)	*Ð*_M_*^e^*
1	1	CH_2_Cl_2_	**G2**	40	1000:1000:100:1	0	256,000 *^f^*	-	-	-
2	1	CH_2_Cl_2_	**G2**	40	2000:2000:200:1	0	512,000*^f^*	-	-	-
3	1	CH_2_Cl_2_	**HG2**	40	500:500:20:1	8	80,300	-	-	-
4	1	THF	**HG2**	60	500:500:25:1	80	6600	6900	17,500	1.5
5	1	THF	**HG2**	60	500:500:100:1	45	3200	3400	10,100	1.3
6	1	THF	**HG2**	60	500:500:50:1	50	5350	5700	17,300	1.5
7	1	THF	**HG2**	60	1000:1000:20:1	75	17,300	17,900	26,800	1.5
8	3	CH_2_Cl_2_	**G2**	40	1000:1000:50:1	100	5500	5900	12,600	2.1
9	3	CH_2_Cl_2_	**G2**	40	1000:1000:100:1	100	2900	2900	8700	1.7

*^a^* General operating conditions: Catalyst = 5.9 µmol, [NB + CDT]_0_ = 1.8 mol·L^−1^; NB and CDT conversion = 100% as determined by a ^1^H NMR analysis (refer to the Experimental Section). *^b^* Conversion in CTA as determined by a ^1^H NMR analysis (refer to the Experimental Section). *^c^* Theoretical molar mass value calculated from *M*_n,theo_ = {(*M*_NB_ × ([NB]_0_ × Conv._NB_) + *M*_CDT_ × ([CDT]_0_ × Conv._CDT_))/([CTA]_0_ × Conv._CTA_)} + *M*_CTA_, with *M*_CDT_ = 162 g·mol^−1^, *M*_NB_ = 94 g·mol^−1^, *M*_CTA**1**_ = 228 g·mol^−1^, and *M*_CTA3_ = 340 g·mol^−1^, on the basis of the formation of only DF (i.e., without taking into account any CNF polymers). *^d^* Experimental molar mass value of the copolymer determined by a ^1^H NMR analysis (refer to the Experimental Section). *^e^* Number-average molar mass (*M*_n,SEC_) and dispersity (*Ð*_M_ = *M*_w_/*M*_n_) values determined by SEC versus polystyrene standards (uncorrected *M*_n_ values) in THF at 30 °C. *^f^* Theoretical molar mass value calculated from *M*_n,theo_ = ([NB]_0_/[**G2**]_0_ × Conv._NB_ × *M*_NB_) + ([CDT]_0_/[**G2**]_0_ × Conv._CDT_ × *M*_CDT_)_,_ with *M*_CDT_ = 162 g·mol^−1^ and *M*_NB_ = 94 g·mol^−1^, on the basis of the formation of only nonfunctional PCOE.

**Table 4:** Macromolecular characteristics of α,ω-bis(dithiocarbonate) P(NB-*co*-CDT) copolymers.

	α,ω-Diepoxide P(NB-*co*-CDT)			α,ω-Bis(dithiocarbonate) P(NB-*co*-CDT)		
Entry	*M*_n,NMR_*^a^* (g·mol^−1^)	*M*_n,sec_*^b^* (g·mol^−^^1^)	*Ð*_M_*^b^*	*M*_n,NMR_*^a^* (g·mol^−1^)	*M*_n,sec_*^b^* (g·mol^−^^1^)	*Ð*_M_*^b^*
1	3400	10,100	1.3	3400	10,200	1.5
2	5700	17,300	1.5	6100	19,100	1.8
3	6600	19,900	1.5	8700	24,700	1.8

*^a^* Experimental molar mass value of the copolymer determined by a ^1^H NMR analysis (refer to the Experimental Section). *^b^* Number-average molar mass (*M*_n,SEC_) and dispersity (*Ð*_M_ = *M*_w_/*M*_n_) values determined by SEC versus polystyrene standards (uncorrected *M*_n_ values) in THF at 30 °C.

**Table 5:** Thermal transitions of the telechelic (co)POs *^a^*.

Entry	Polymer	CTA/Chain-End Group	*M*_n,NMR_*^d^* (g·mol^−1^)	*T*_g_*^a^* (°C)	*T*_m_*^a^* (°C)	∆*Hfus ^a^* (J/g)	*T*_c_*^a^* (°C)
1	PCOE	1/epoxide	3900	*n.o.*	58	21	45
2	PCOE	3/oxetane	3700	−45	54	23	41
3	P(NB-*co*-CDT)	1/epoxide	6900	−75	5	5	−14
4	P(NB-*co*-CDT)	3/oxetane	5900	−72	4	5	−16
5	P(NB-*co*-CDT)	-/dithiocarbonate	3400	−75	5	5	−14
6	P(NB-*co*-CDT)	-/dithiocarbonate	8100	−74	5	5	−12
7	P(NB-*co*-CDT)	-/dithiocarbonate	8700	*n.o.*	4	5	−10

*^a^* Glass transition, melting, and crystallization temperatures and melting enthalpy as determined by DSC analyses (second heating scan) (refer to the Experimental Section)*. n.o.*, not observed.

**Table 6:** Newtonian viscosity of telechelic P(NB-*co*-CDT) copolymers.

Entry	Telechelic Copolymer	CTA/Chain-End Group	*M*_n,NMR_*^a^* (g·mol^−1^)	Newtonian Viscosity at 25 °C *^a^* (Pa·s)
1	P(COE-*co*-NB)	1/epoxide	6600	750 ± 50
2	P(COE-*co*-NB^COOMe^)	1/epoxide	22,700	800 ± 50
3	P(NB-*co*-CDT)	1/epoxide	6900	14 ± 3
4	P(NB-*co*-CDT)	3/oxetane	5900	13 ± 5
5	P(NB-*co*-CDT)	-/dithiocarbonate	3400	14 ± 5
6	P(NB-*co*-CDT)	-/dithiocarbonate	6100	15 ± 5
7	P(NB-*co*-CDT)	-/dithiocarbonate	8700	17 ± 3

*^a^* Measured using an ARES **G2** TA instrument (refer to the Experimental Section).

## Supplementary Materials

The following are available online at http://www.mdpi.com/2073-4360/10/11/1241/s1, The Supporting Information includes complementary macromolecular and characterization data, including 1H, 13C{1H}, and 2D NMR and FTIR spectra, X-ray structures of CTAs, and polymers.

## References

[1] Goethals E.J. (1989). Telechelic Polymers: Synthesis and Applications.

[2] Odian G. (2004). Principles of Polymerization.

[3] Hanik N., Kilbinger A.F.M., Grubbs R.H., Wenzel A.G., O’Leary D.J., Khosravi E. (2015). Telechelic Polymers. Handbook of Metathesis.

[4] Guillaume S.M. (2017). Handbook of Telechelic Polyesters, Polycarbonates and Polyethers.

[5] Chung T.C. (2002). Functionalization of Polyolefins.

[6] Yanjarappa M.J., Sivaram S. (2002). Recent developments in the synthesis of functional poly (olefin)s. Prog. Polym. Sci..

[7] Boaen N.K., Hillmyer M.A. (2005). Post-polymerization functionalization of polyolefins. Chem. Soc. Rev..

[8] Amin S.B., Marks T.J. (2008). Versatile pathways for in situ polyolefin functionalization with heteroatoms: catalytic chain transfer. Angew. Chem. Int. Ed..

[9] Hilf S., Kilbinger A.F.M. (2009). Functional end groups for polymers prepared using ring-opening metathesis polymerization. Nat. Chem..

[10] Franssen N.M.G., Reek J.N.H., de Bruin B. (2013). Synthesis of functional ‘polyolefins’: State of the art and remaining challenges. Chem. Soc. Rev..

[11] Bielawski C.W., Hillmyer M.A., Grubbs R.H., Wenzel A.G., O’Leary D.J., Khosravi E. (2015). Telechelic Polymers from Olefins Meztathesis Methodologies. Handbook of Metathesis.

[12] Hutley T.J., Ouederni M., AlMa’adeed M.A.A., Krupa I. (2016). Polyolefin Compounds and Materials—Fundamentals and Industrial Applications.

[13] Guillaume S.M. (2018). Advances in the synthesis of silyl-modified polymers (SMPs). Polym. Chem..

[14] Wang Y., Hillmyer M. (2015). Hydroxy-telechelic poly (ethylene-co-isobutylene) as a soft segment for thermoplastic polyurethanes. Polym. Chem..

[15] Martinez H., Hillmyer M. (2014). Carboxy-telechelic polyolefins in cross-linked elastomers. Macromolecules.

[16] Amendt M.A., Pitet L.M., Moench S., Hillmyer M. (2012). Reactive triblock polymers from tandem ring-opening polymerization for nanostructured vinyl thermosets. Polym. Chem..

[17] LPitet M., Hillmyer M. (2011). Carboxy-telechelic polyolefins by ROMP using maleic acid as a chain transfer agent. Macromolecules.

[18] Pitet L.M., Chamberlain M.M., Hauser A.W., Hillmyer M. (2010). Synthesis of linear, H-shaped, and arachnearm block copolymers by tandem ring-opening polymerizations. Macromolecules.

[19] Thomas M.R., Grubbs R.H. (2010). Synthesis of telechelic polyisoprene via ring-opening metathesis polymerization in the presence of chain transfer agent. Macromolecules.

[20] Pitet L.M., Hillmyer M. (2009). Combining Ring-Opening Metathesis Polymerization and Cyclic Ester Ring-Opening Polymerization To Form ABA Triblock Copolymers from 1,5-Cyclooctadiene and d,l-Lactide. Macromolecules.

[21] Ji S., Hoye T.R., Macosko C.W. (2008). Diamino telechelic polybutadienes for solventless styrene–butadiene–styrene (SBS) triblock copolymer formation. Polymer.

[22] Ji S., Hoye T.R., Macosko C.W. (2004). Controlled synthesis of high molecular weight telechelic polybutadienes by ring-opening metathesis polymerization. Macromolecules.

[23] Scherman O.A., Rutenberg I.M., Grubbs R.H. (2003). Direct synthesis of soluble, end-functionalized polyenes and polyacetylene block copolymers. J. Am. Chem. Soc..

[24] Bielawski C.W., Scherman O.A., Grubbs R.H. (2001). Highly efficient syntheses of acetoxy-and hydroxy-terminated telechelic poly (butadiene) s using ruthenium catalysts containing N-heterocyclic ligands. Polymer.

[25] Bielawski C.W., Benitez D., Morita T., Grubbs R.H. (2001). Synthesis of end-functionalized poly (norbornene) s via ring-opening metathesis polymerization. Macromolecules.

[26] Gibson V.C., Okada T. (2000). Synthesis of end-functionalized polynorbornenes and polynorbornanes via metathesis: novel macromonomers for polycondensation reactions. Macromolecules.

[27] Morita T., Maughon B.R., Bielawski C.W., Grubbs R.H. (2000). A Ring-Opening Metathesis Polymerization (ROMP) Approach to Carboxyl- and Amino-Terminated Telechelic Poly(butadiene)s. Macromolecules.

[28] Maughon B.R., Morita T., Bielawski C.W., Grubbs R.H. (2000). Synthesis of cross-linkable telechelic poly (butenylene) s derived from ring-opening metathesis polymerization. Macromolecules.

[29] Hillmyer M.A., Nguyen S.B.T., Grubbs R.H. (1997). Utility of a ruthenium metathesis catalyst for the preparation of end-functionalized polybutadiene. Macromolecules.

[30] Fraser C., Hillmyer M.A., Gutierrez E., Grubbs R.H. (1995). Degradable Cyclooctadiene/Acetal Copolymers: Versatile Precursors to 1,4-Hydroxytelechelic Polybutadiene and Hydroxytelechelic Polyethylene. Macromolecules.

[31] Hillmyer M.A., Grubbs R.H. (1995). Chain transfer in the ring-opening metathesis polymerization of cyclooctadiene using discrete metal alkylidenes. Macromolecules.

[32] Chung T.C., Chasmawala M. (1992). Synthesis of telechelic 1, 4-polybutadiene by metathesis reactions and borane monomers. Macromolecules.

[33] Saetung N., Campistron I., Pascual S., Pilard J.-F., Fontaine L. (2011). One-Pot Synthesis of Natural Rubber-Based Telechelic cis-1,4-Polyisoprenes and Their Use To Prepare Block Copolymers by RAFT Polymerization. Macromolecules.

[34] Annunziata L., Fouquay S., Michaud G., Simon F., Guillaume S.M., Carpentier J.-F. (2013). Mono- and di-cyclocarbonate telechelic polyolefins synthesized from ROMP using glycerol carbonate derivatives as chain-transfer agents. Polym. Chem..

[35] Diallo A.K., Annunziata L., Fouquay S., Michaud G., Simon F., Brusson J.-M., Guillaume S.M., Carpentier J.-F. (2014). Ring-opening metathesis polymerization of cyclooctene derivatives with chain transfer agents derived from glycerol carbonate. Polym. Chem..

[36] Annunziata L., Diallo A.K., Fouquay S., Michaud G., Simon F., Brusson J.-M., Carpentier J.-F., Guillaume S.M. (2014). α, ω-Di (glycerol carbonate) telechelic polyesters and polyolefins as precursors to polyhydroxyurethanes: an isocyanate-free approach. Green Chem..

[37] Diallo A.K., Michel X., Fouquay S., Michaud G., Simon F., Brusson J.-M., Guillaume S.M., Carpentier J.-F. (2015). α-Trialkoxysilyl functionalized polycyclooctenes synthesized by chain-transfer ring-opening metathesis polymerization. Macromolecules.

[38] Michel X., Fouquay S., Michaud G., Simon F., Brusson J.-M., Carpentier J.-F., Guillaume S.M. (2016). α, ω-Bis (trialkoxysilyl) difunctionalized polycyclooctenes from ruthenium-catalyzed chain-transfer ring-opening metathesis polymerization. Polym. Chem..

[39] Vanbiervliet E., Fouquay S., Michaud G., Simon F., Carpentier J.-F., Guillaume S.M. (2017). From Epoxide to Cyclodithiocarbonate Telechelic Polycyclooctene through Chain-Transfer Ring-Opening Metathesis Polymerization (ROMP): Precursors to Non-Isocyanate Polyurethanes (NIPUs). Macromolecules.

[40] Michel X., Fouquay S., Michaud G., Simon F., Brusson J.-M., Carpentier J.-F., Guillaume S.M. (2017). Tuning the properties of α, ω-bis (trialkoxysilyl) telechelic copolyolefins from ruthenium-catalyzed chain-transfer ring-opening metathesis polymerization (ROMP). Polym. Chem..

[41] Michel X., Fouquay S., Michaud G., Simon F., Brusson J.-M., Carpentier J.-F., Guillaume S.M. (2017). Simple access to alkoxysilyl telechelic polyolefins from ruthenium-catalyzed cross-metathesis depolymerization of polydienes. Eur. Polym. J..

[42] Chauveau C., Vanbiervliet E., Fouquay S., Michaud G., Simon F., Carpentier J.-F., Guillaume S.M. (2018). Azlactone Telechelic Polyolefins as Precursors to Polyamides: A Combination of Metathesis Polymerization and Polyaddition Reactions. Macromolecules.

[43] Zhang Y., Sudo A., Endo T. (2008). Syntheses of bisphenol-type oligomers having five-membered dithiocarbonate groups at the terminals and their application as accelerators to epoxy-amine curing system. J. Polym. Sci. A Polym. Chem..

[44] Rauleder G., Roxo B., Waldmann H., Bottenbruch L., Traenckner H.J., Gau W. (1985). Method for the preparation and separation of polyglycidyl compounds.

[45] Otton J., Colleuille Y., Varagnat J. (1980). Metathesis of functionalised olefins: homogeneous cross-metathesis of cycloolefin and ethylenic esters. J. Mol. Cat..

[46] Darensbourg D.J., Horn A., Moncadra A.I. (2010). A facile catalytic synthesis of trimethylene carbonate from trimethylene oxide and carbon dioxide. Green Chem..

[47] Kobayashi T. (2011). Lipase-catalyzed syntheses of sugar esters in non-aqueous media. Biotechnol. Lett..

[48] Rouillard H. (January 2012). Etude de Résolutions Catalysées par des Lipases sous Irradiation Micro-Onde. Ph.D. Dissertation.

[49] Martinez H., Ren N., Matta M.E., Hillmyer M.A. (2014). Ring-opening metathesis polymerization of 8-membered cyclic olefins. Polym. Chem..

