# Supplementary material for: α,ω-Epoxide, Oxetane, and Dithiocarbonate Telechelic Copolyolefins: Access by Ring-Opening Metathesis/Cross-Metathesis Polymerization (ROMP/CM) of Cycloolefins in the Presence of Functional Symmetric Chain-Transfer Agents
Source: Polymers (Basel). 2018 Nov 9;10(11):1241. doi: 10.3390/polym10111241 (PMC6401821; doi:10.3390/polym10111241)
Supplement: Supplementary file 1 [file polymers-10-01241-s001.pdf]

polymers

MDPI

Supplementary

α,ω-Epoxide, Oxetane and Dithiocarbonate Telechelic Copolyolefins: Access by Ring-Opening Metathesis/Cross-Metathesis Polymerization (ROMP/CM) of Cycloolefins in the Presence of Functional Symmetric Chain-Transfer Agents

Elise Vanbiervliet,[a] Stéphane Fouquay,[b] Guillaume Michaud,[c] Frédéric Simon,[c] Jean-François Carpentier (ORCID: 0000-0002-9160-7662),[a,1] and Sophie M. Guillaume (ORCID: 0000-0003-2917-8657)[a,*]

[a] Univ Rennes, CNRS, ISCR (Institut des Sciences Chimiques de Rennes) – UMR 6226, F-35000 Rennes, France
[b] BOSTIK S.A., 253, Avenue du Président Wilson, F-93211 La Plaine Saint-Denis, France
[c] BOSTIK, ZAC du Bois de Plaisance, 101, Rue du Champ Cailloux, F-60280 Venette, France
[*] Corresponding authors: jean-francois.carpentier@univ-rennes1.fr; sophie.guillaume@univ-rennes1.fr

Table, Schemes and Figures captions

Table S1. NMR and FTIR spectroscopic characteristics of the α,ω-diepoxide telechelic P(NB-*co*-CDT) prepolymers and the resulting α,ω-bis(dithiocarbonate) P(NB-*co*-CDT) analogues.

Figure S1. ORTEP representation of the molecular solid-state structure of CTA **2**. Ellipsoids drawn at the 50% probability level. H atoms are omitted for clarity.

Figure S2. ^{1}H NMR spectrum (400 MHz, DMSO-d_6, 25 °C) of CTA **2**; (*: residual solvents δ (ppm) 3.31 H_2O, 1.25, 4.20 ethanol).

Figure S3. ^{13}C{^{1}H} NMR spectrum (100 MHz, DMSO-d_6, 25 °C) of CTA **2**; (*: residual solvents δ (ppm) 13.9; 61.1 diethylether; 18.6, 56.1 ethanol).

Figure S4. ^{1}H NMR spectrum (400 MHz, DMSO-d_6, 25 °C) of CTA **3**; (*: residual solvent δ (ppm) 3.31 H_2O).

Figure S5. ^{13}C{^{1}H} NMR spectrum (100 MHz, DMSO-d_6, 25 °C) of CTA **3**.

Figure S6. ^{1}H NMR spectrum (500 MHz, $CDCl_3$, 25 °C) of the copolymer sample prepared by ROMP/CM of COE/NB (50:50) in the presence of **G2**/CTA **1** in CH_2Cl_2 (Table 2, entry 1). (*: residual solvents: δ (ppm) 1.59 H_2O, 0.07 grease).

Figure S7. ^{13}C NMR spectrum (125 MHz, $CDCl_3$, 25 °C) of the copolymer sample prepared by ROMP/CM of COE/NB (50:50) in the presence of **G2**/CTA **1** (Table 2, entry 1). (*: residual solvents: δ (ppm) 1.2 grease).

Figure S8. ^{1}H NMR spectrum (500 MHz, $CDCl_3$, 25 °C) of the copolymer sample prepared by ROMP/CM of NB/CDT (50:50) in the presence of **HG2**/CTA **1** in CH_2Cl_2 (Table 3, entry 3). (*: residual solvents: δ (ppm) 1.53 H_2O, 0.07 grease).

Figure S9. ^{1}H NMR spectrum (500 MHz, $CDCl_3$, 25 °C) of the copolymer sample prepared by ROMP/CM of NB/CDT (50:50) in the presence of **HG2**/CTA **1** in THF (Table 3, entry 4), a) after dialysis in THF, and b) before dialysis in THF (*: residual solvents: δ (ppm) 1.53 H_2O, 0.07 grease).

Figure S10. ^{13}C NMR spectrum (125 MHz, $CDCl_3$, 25 °C) of the copolymer sample prepared by ROMP/CM of NB/CDT (50:50) in the presence of **HG2**/CTA **1** in THF (Table 3, entry 4) (δ (ppm) 0.07 grease).

Figure S11. 2D COSY ^{1}H–^{1}H NMR spectrum (400 MHz, $CDCl_3$, 25 °C) of the copolymer sample prepared by ROMP/CM of NB/CDT (50:50) in the presence of **G2**/CTA **3** (Table 3, entry 8).

Figure S11. 2D COSY ^{1}H–^{1}H NMR spectrum (400 MHz, $CDCl_3$, 25 °C) of the copolymer sample prepared by ROMP/CM of NB/CDT (50:50) in the presence of **G2**/CTA **3** (Table 3, entry 6).

Figure S12. FTIR spectrum of the α,ω-bis(dithiocarbonate) P(NB-*co*-CDT) copolymer sample prepared by dithiocarbonatation of the α,ω-diepoxide telechelic P(NB-*co*-CDT) prepolymer (Table 4, entry 1).

Figure S13. ^{1}H NMR spectrum (500 MHz, $CDCl_3$, 25 °C) of the CNF copolymer isolated from the α,ω-bis(dithiocarbonate) P(NB-*co*-CDT) crude copolymers. (*: δ (ppm) 0.07 residual grease) (Table 4, entry 1).

Table S1. NMR and FTIR spectroscopic characteristics of the α,ω-diepoxide telechelic P(NB-*co*-CDT) prepolymers and the resulting α,ω-bis(dithiocarbonate) P(NB-*co*-CDT) analogues.

^{1}H NMR (400 MHz, 23 °C, $CDCl_3$)	(δ ppm)	2.67; 2.86; 3.24; 3.98; 4. 45 (**A61**)	3.59; 4.49; 5.37
	Assignments	a ; a ; b ; c ; c	a'; c' ; b'
	Integrations	1 1 1 1 1	2 2 -[b]
		Figure S9	Figure 5
$^{13}C\{^{1}H\}$ NMR (100 MHz, 23 °C, $CDCl_3$)	(δ ppm)	38.2 ; 49.6 ; 64.9 ; 166.6	210.9 ; 166.0 ; 87.7 ; 53.6 ; 31.0
	Assignments	a ; b ; c ; d	g' ; d' ; b' ; c' ; a'
		Figure S10	Figure 6
FTIR	(σ cm^{-1})	-	1190 ($\upsilon_{C=S}$) ; 1519 ($\upsilon_{C=O}$)<br>Figure S13

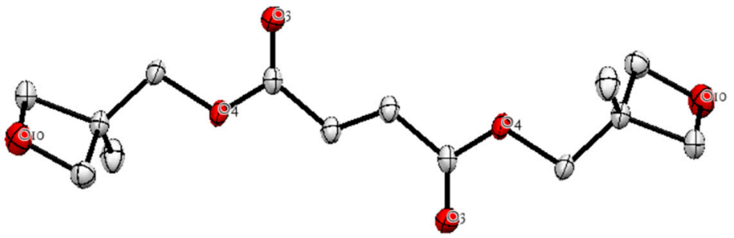

Figure S1. ORTEP representation of the molecular solid-state structure of CTA **2**. Ellipsoids drawn at the 50% probability level. H atoms are omitted for clarity.

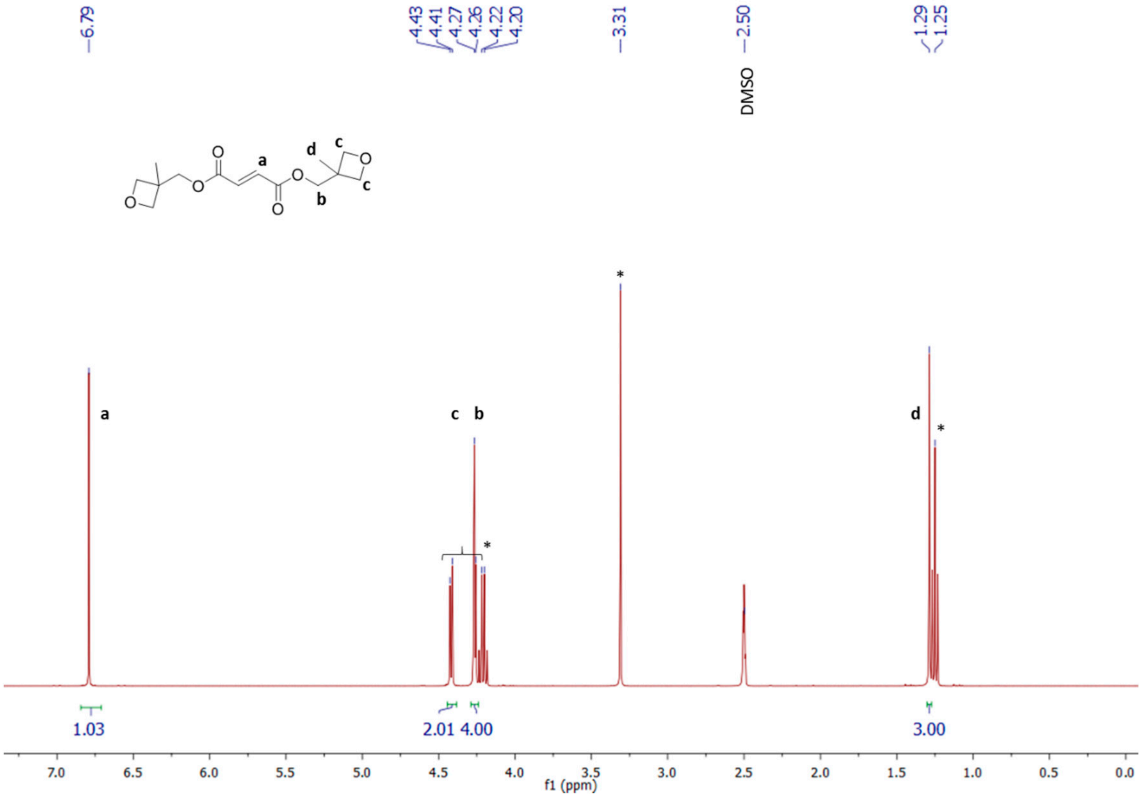

Figure S2. ^{1}H NMR spectrum (400 MHz, DMSO-*d*6, 25 °C) of CTA **2**; (*: residual solvents δ (ppm) 3.31 H_2O, 1.25, 4.20 ethanol).

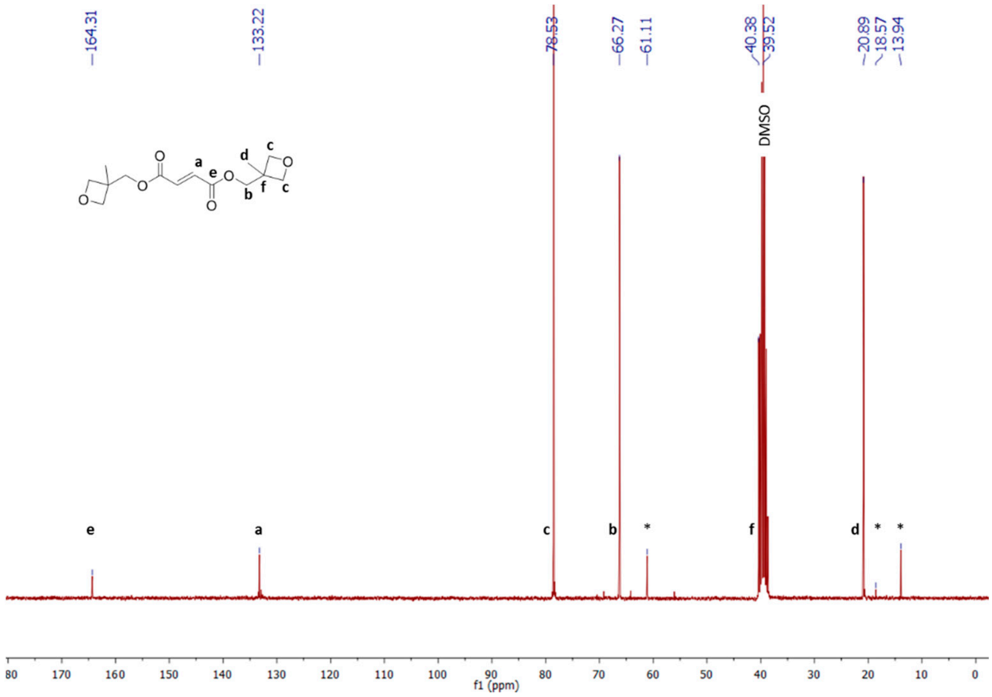

Figure S3. ^{13}C{^{1}H} NMR spectrum (100 MHz, DMSO-*d*6, 25 °C) of CTA **2**; (*: residual solvents δ (ppm) 13.9; 61.1 diethylether; 18.6, 56.1 ethanol).

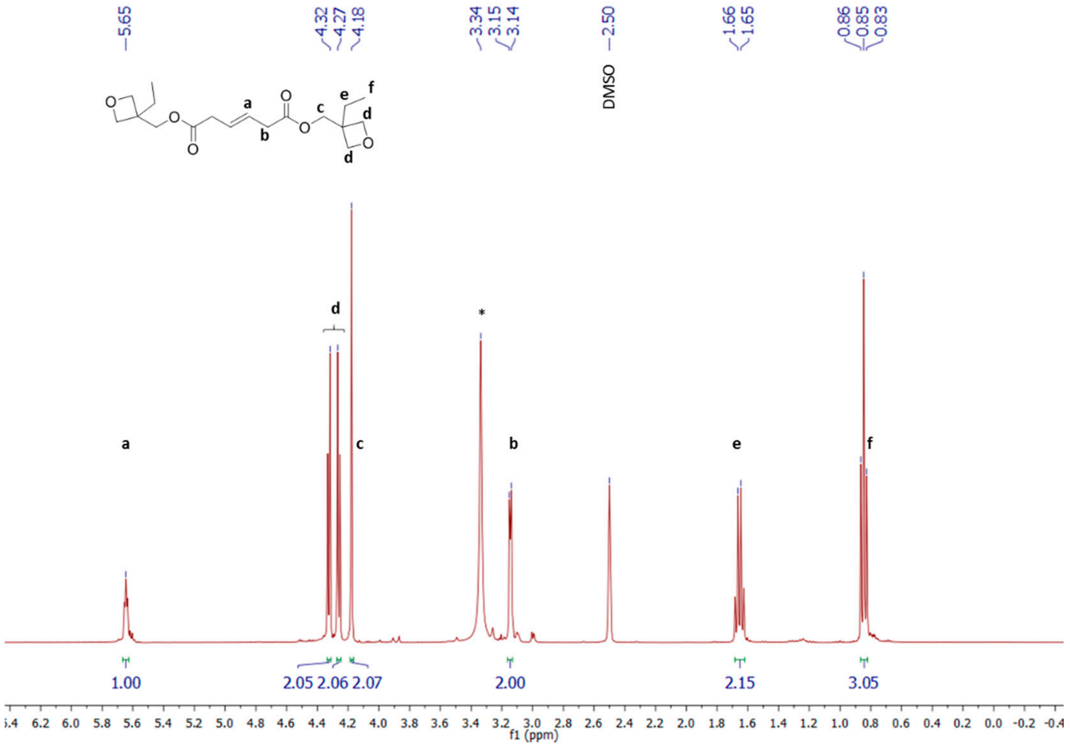

Figure S4. ^1H NMR spectrum (400 MHz, DMSO-d_6, 25 °C) of CTA **3**; (*: residual solvent δ (ppm) 3.31 H_2O).

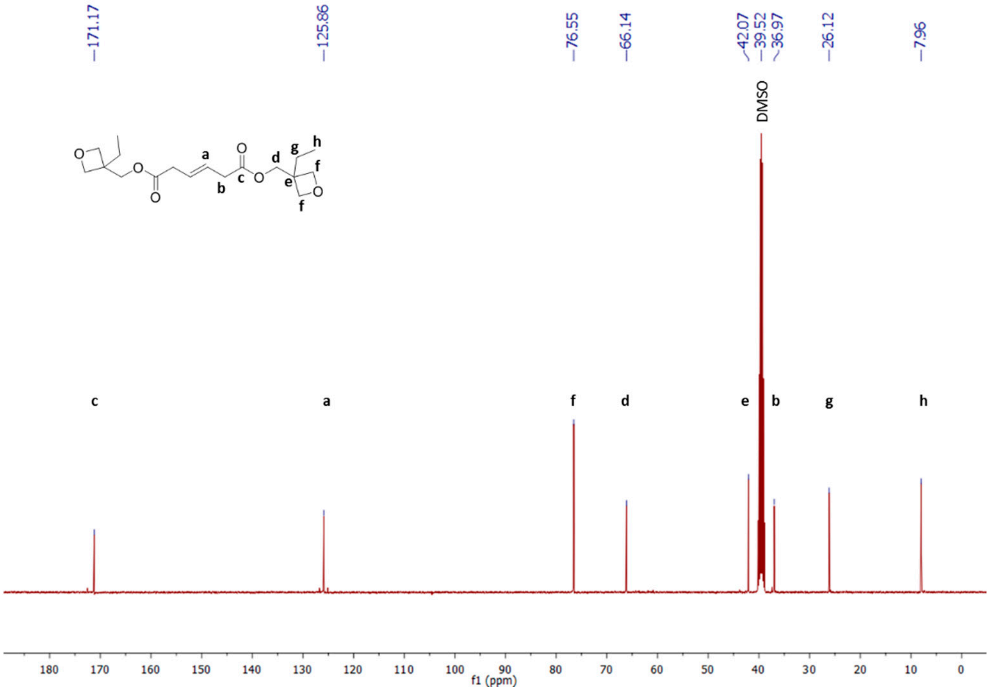

Figure S5. $^{13}C\{^1H\}$ NMR spectrum (100 MHz, DMSO-d_6, 25 °C) of CTA **3**.

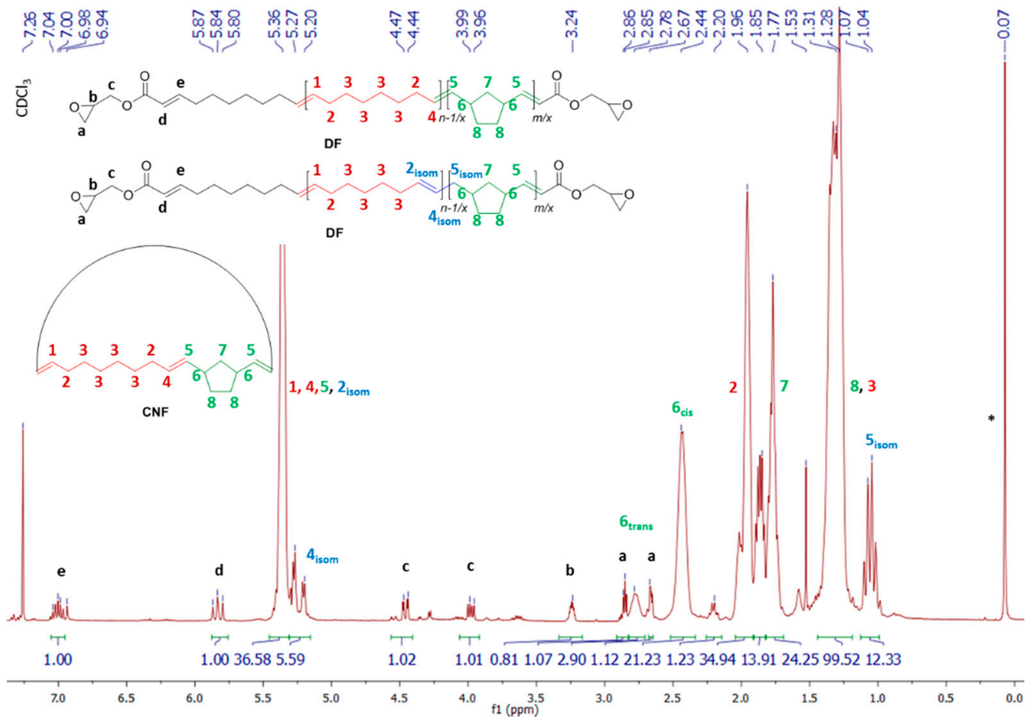

Figure S6. ^{1}H NMR spectrum (500 MHz, $CDCl_3$, 25 °C) of a copolymer sample prepared by ROMP/CM of COE/NB (50:50) in the presence of **G2**/CTA **1** in CH_2Cl_2 (Table 2, entry 1). (*: residual solvents: δ (ppm) 1.59 H_2O, 0.07 grease).

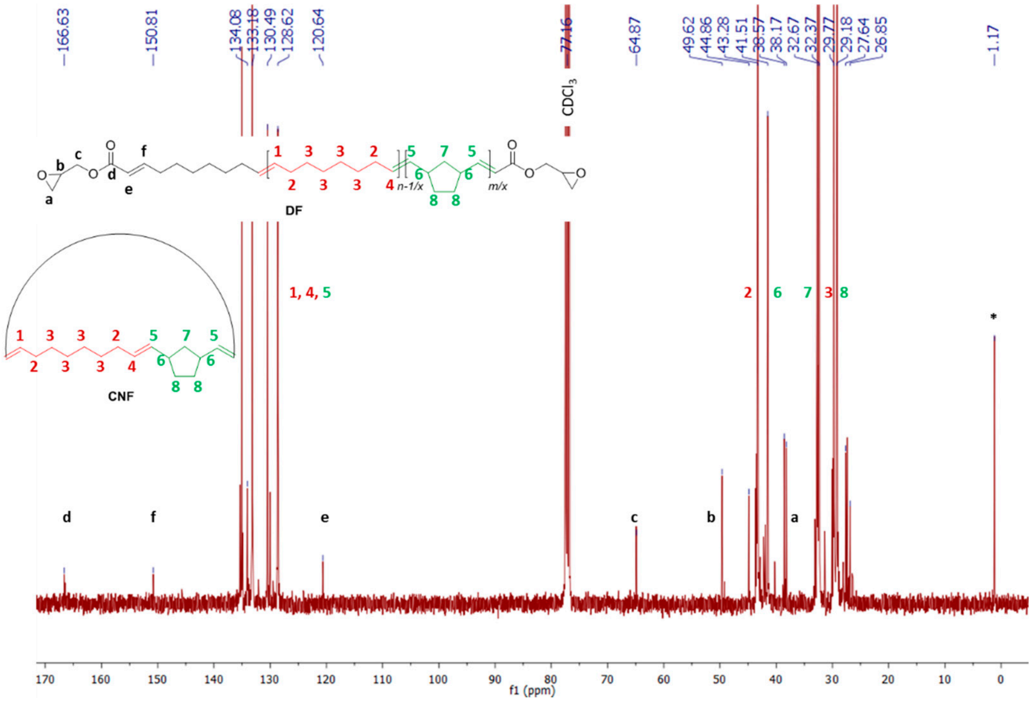

Figure S7. ^{13}C NMR spectrum (125 MHz, $CDCl_3$, 25 °C) of a copolymer sample prepared by ROMP/CM of COE/NB (50:50) in the presence of **G2**/CTA **1** (Table 2, entry 1). (*: residual solvents: δ (ppm) 1.2 grease).

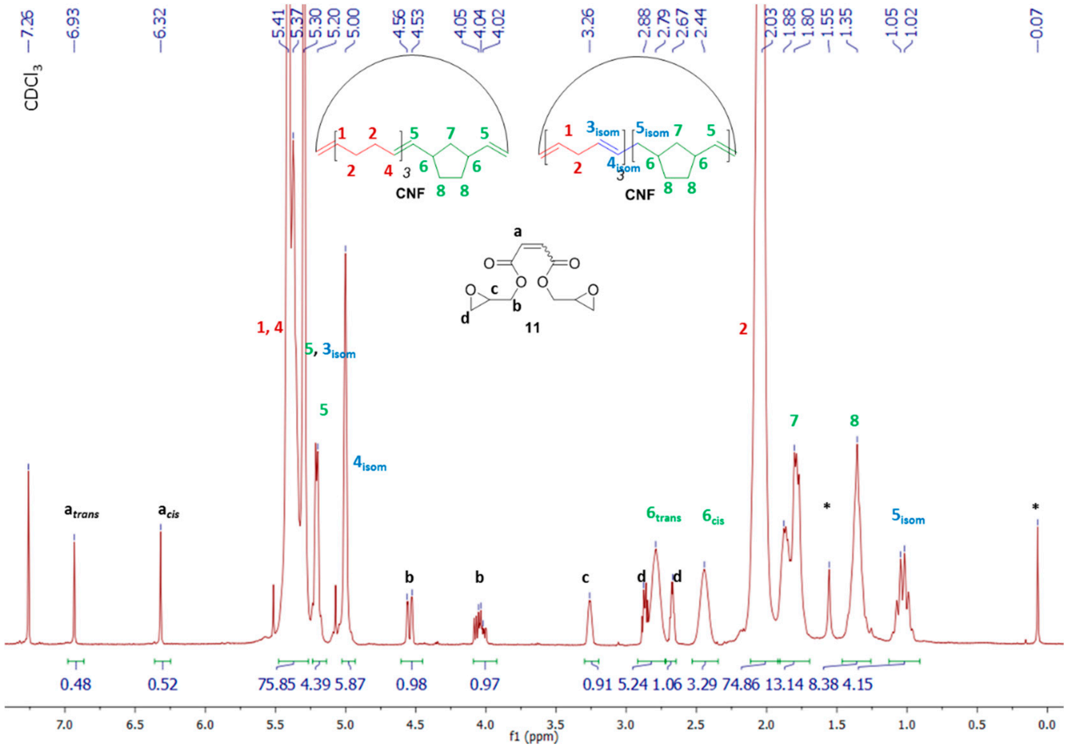

Figure S8. ^{1}H NMR spectrum (500 MHz, $CDCl_3$, 25 °C) of a copolymer sample prepared by ROMP/CM of NB/CDT (50:50 mol/mol) in the presence of **HG2**/CTA **1** in CH_2Cl_2 (Table 3, entry 3). (*: residual solvents: δ (ppm) 1.53 H_2O, 0.07 grease).

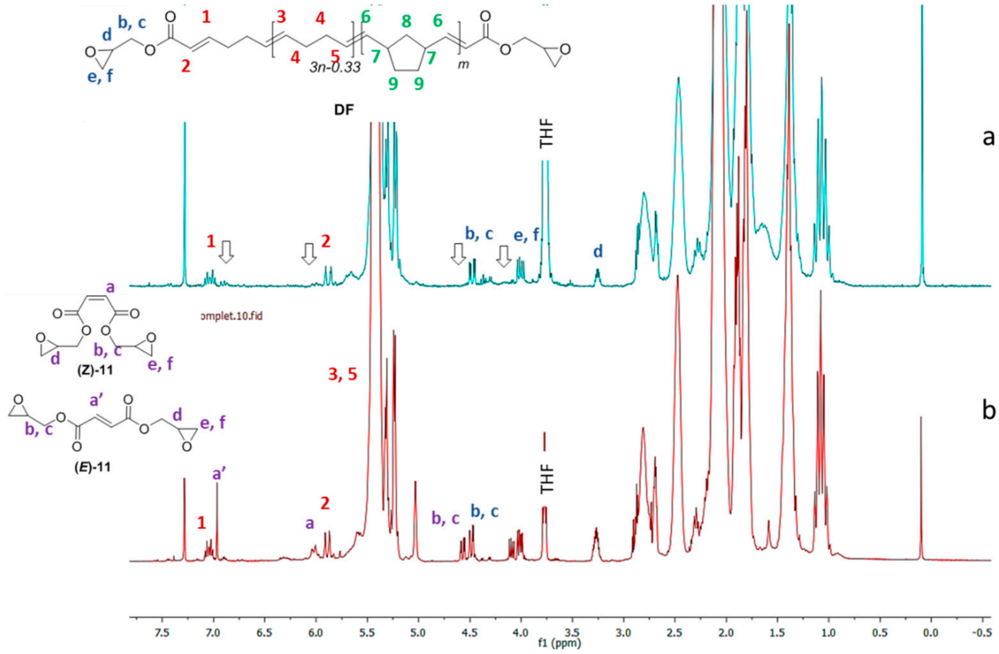

Figure S9. ^{1}H NMR spectrum (500 MHz, $CDCl_3$, 25 °C) of a copolymer sample prepared by ROMP/CM of NB/CDT (50:50 mol/mol) in the presence of **HG2**/CTA **1** in THF (Table 3, entry 4), a) after dialysis in THF, and b) before dialysis in THF (*: residual solvents: δ (ppm) 1.53 H_2O, 0.07 grease).

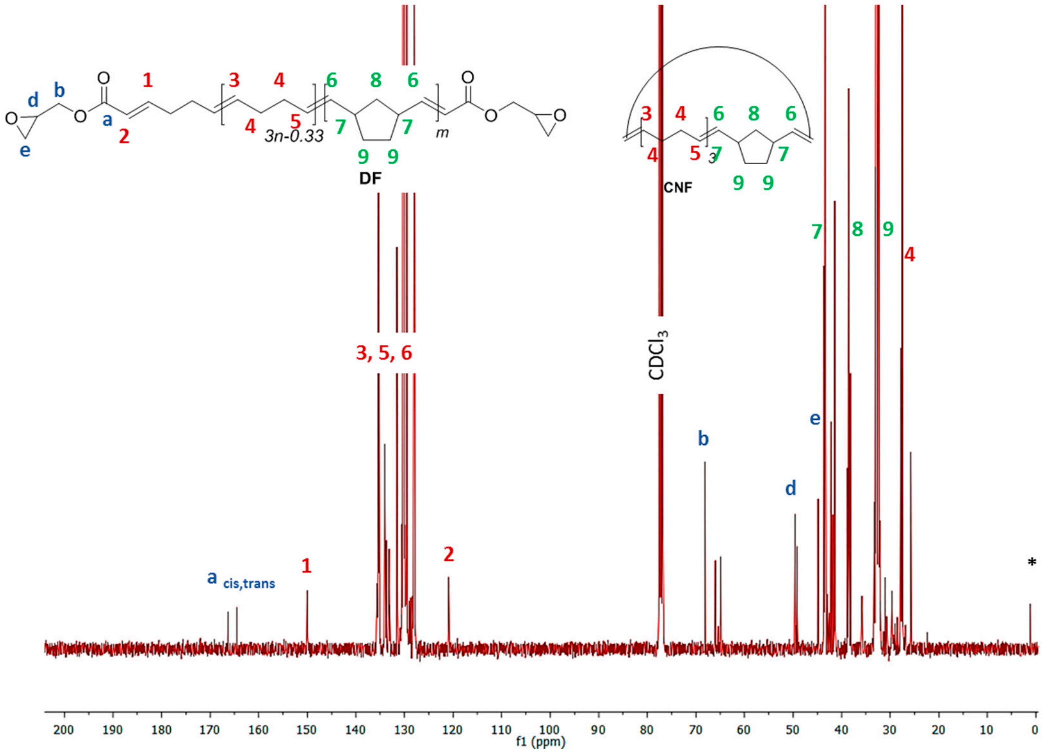

Figure S10. ^{13}C NMR spectrum (125 MHz, $CDCl_3$, 25 °C) of a copolymer sample prepared by ROMP/CM of NB/CDT (50:50 mol/mol) in the presence of **HG2**/CTA **1** in THF (Table 3, entry 4) (δ (ppm) 0.07 grease).

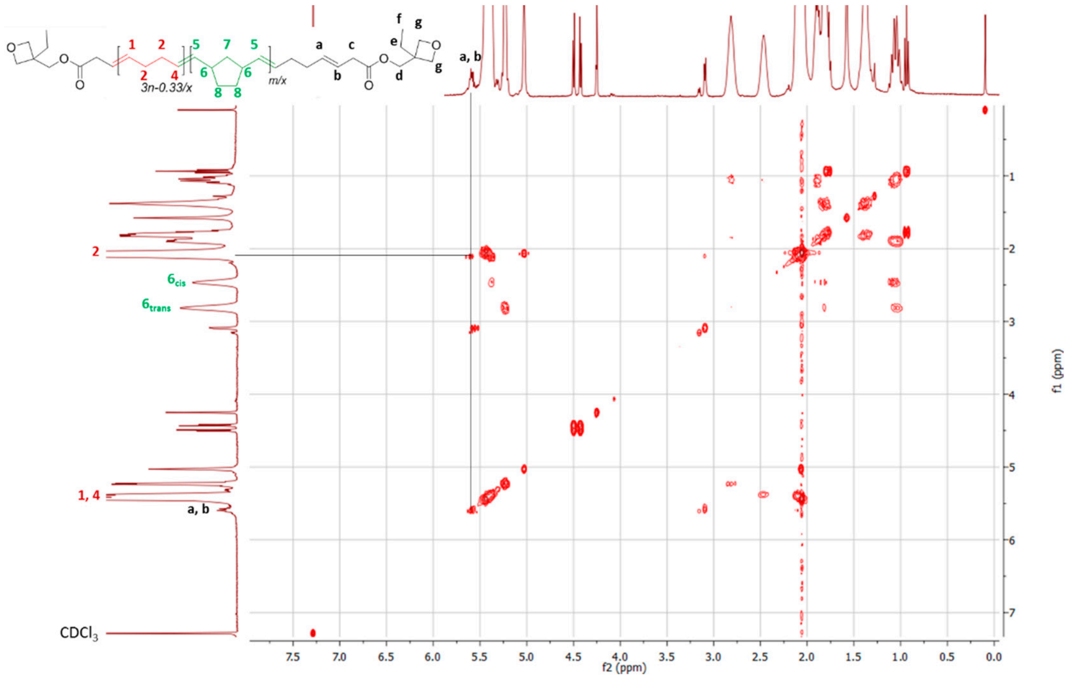

Figure S11. 2D COSY ^{1}H–^{1}H NMR spectrum (400 MHz, $CDCl_3$, 25 °C) of a copolymer sample prepared by ROMP/CM of NB/CDT (50:50 mol/mol) in the presence of **G2**/CTA **3** (Table 3, entry 8).

.

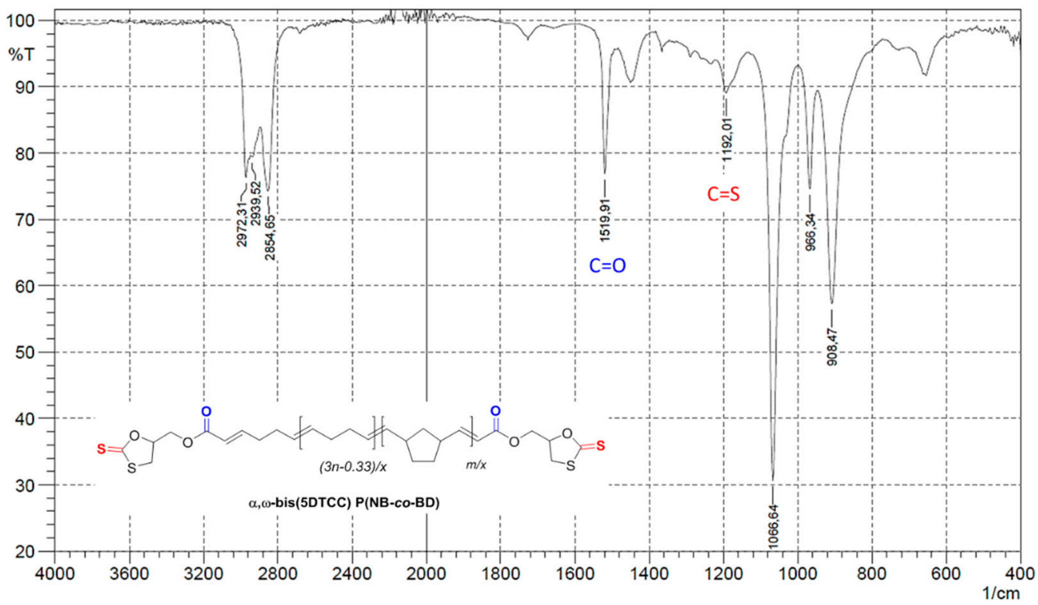

Figure S12. FTIR spectrum of an α,ω-bis(dithiocarbonate) P(NB-*co*-CDT) copolymer sample prepared by dithiocarbonatation of an α,ω-diepoxide telechelic P(NB-*co*-CDT) prepolymer (Table 4, entry 1).

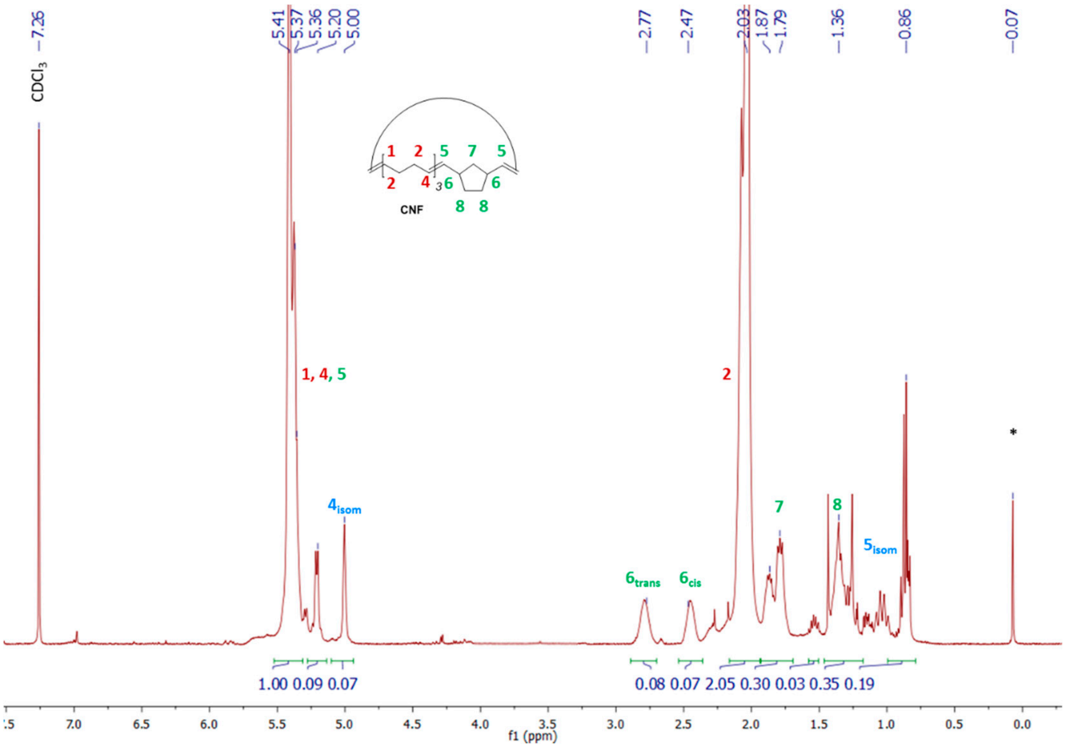

Figure S13. ^{1}H NMR spectrum (500 MHz, $CDCl_3$, 25 °C) of a CNF copolymer isolated from an α,ω-bis(dithiocarbonate) P(NB-*co*-CDT) crude copolymers. (*: δ (ppm) 0.07 residual grease) (Table 4, entry 1).

© 2018 by the authors. Submitted for possible open access publication under the terms and conditions of the Creative Commons Attribution (CC BY) license (http://creativecommons.org/licenses/by/4.0/).
